# Cross-Species and Cell-Type-Specific H3K27ac Dynamics Link Brain Aging to Alzheimer’s Disease Genes

**DOI:** 10.3390/cells15212007

**Published:** 2026-11-04

**Authors:** Ching-Thong Choo, Zhen-Kai Ngian, Chin-Tong Ong

**Affiliations:** 1 Temasek Life Sciences Laboratory, Singapore 117604, Singapore; choo_ct@tll.org.sg (C.-T.C.);; 2 Department of Biological Sciences, National University of Singapore, Singapore 117543, Singapore

**Keywords:** H3K27ac, NeuN− cells, aging brain, Alzheimer’s disease

## Abstract

Active H3K27ac marks are dynamically remodelled during brain aging, but their cell-type-specific regulatory roles remain unclear. We integrated genome-wide H3K27ac profiles and transcriptomes from aging *Drosophila* heads, mouse NeuN+ forebrain neurons, and human NeuN+/NeuN− frontal cortex cells. In *Drosophila*, age-gained differential (Δ) H3K4me1 and H3K27ac peaks were associated with RNA processing and immune response genes, respectively, suggesting distinct regulatory programs. Despite limited biological replicates, mouse analyses revealed a strong association between promoter-associated ΔH3K27ac and synaptic gene expression in young neurons. Age-related gene-body H3K27ac loss coincided with increased expression of genes involved in GTPase signalling and vesicular transport. In humans (n = 29), NeuN− cells exhibited more age-associated ΔH3K27ac alterations than NeuN+ cells. Genes associated with age-gained peaks were enriched for gliogenesis and amyloid precursor protein metabolic processes, while age-related transcriptional changes associated positively with super-enhancer activity. Comparison with pan-tissue aging signatures revealed shared and cell-type-specific age-related ΔH3K27ac patterns in NeuN+ and NeuN− cells. Integrative analyses showed that age-gained ΔH3K27ac peaks in NeuN− cells overlapped with AD-associated ΔH3K27ac peaks, differentially expressed AD genes, and AD GWAS-linked variants, including those mapped to *BIN1* and *CR1*. These findings highlight extensive H3K27ac remodelling in aging NeuN− cells and its convergence with AD-associated molecular alterations.

## 1. Introduction

Enhancers regulate the spatiotemporal specificity of gene expression and are typically marked by H3K4me1 in a poised state and H3K27ac in an active state [1]. Age-associated changes in H3K27ac influence tissue-specific transcriptional programs. For example, endometrial aging is associated with H3K27ac loss and reduced progesterone receptor expression [2], whereas increased H3K27ac drives the upregulation of extracellular matrix genes in aged skeletal muscle [3]. Similarly, in aging mouse bone marrow mesenchymal stem cells, reduced enhancer H3K27ac is accompanied by decreased expression of the IGF2BP2 (Insulin-like growth factor 2 mRNA-binding protein 2) [4].

In the mouse brain, H3K27ac occupancy decreases at synaptic and ion transport genes in aging forebrain NeuN+ neurons but increases at interferon-responsive genes in the cerebellum [5,6]. Paradoxically, reduced gene-body H3K27ac levels at inflammaging genes has been linked to their upregulation in aged brain tissue [7], highlighting the context-dependent regulatory roles of H3K27ac in aging. Notably, non-mutagenic double-stranded DNA breaks (DSB) induce erosion of H3K27ac landscape in mouse cells and accelerate aging phenotypes, a process that can be reversed by Oct4-Sox2-KLF4-mediated rejuvenation [8]. Moreover, targeted depletion of H3K27ac peaks near IGF2BP3 gene in HL-60 cells induces the expression of senescence-associated markers [9]. Together, these findings suggest that H3K27ac marks may both reflect and drive biological aging. To investigate the age-related dynamics of H3K27ac and their regulatory effects on gene expression across brain tissues and species, we analysed H3K27ac landscapes in aging *Drosophila* heads, mouse NeuN+ forebrain neurons [6], and human NeuN+ and NeuN− frontal cortex cells [10]. We further integrated the human H3K27ac datasets with multi-omics datasets from Alzheimer’s disease (AD) and pan-tissue histone signatures [11] to examine whether age-dependent H3K27ac changes are associated with AD-related molecular alterations and broader epigenetic patterns.

## 2. Results

### 2.1. ΔH3K4me1 and ΔH3K27ac Peaks Associate with Distinct Pathways in Aging Drosophila Head Tissues

In *Drosophila*, housekeeping (hk) and developmental (d) genes are regulated by distinct enhancer-core promoter (CP) specificities [12]. hkCP enhancers are marked by H3K4me3 near transcription start site (TSS; <0.2 kb), whereas dCP enhancers are enriched for H3K4me1 within the intronic and intergenic regions [13]. Adult male *w^1118^* flies, with a lifespan of approximately 60 days [14], enable time-dependent transcriptomic analyses of head tissues during aging (Table S1A) [15]. RNA-sequencing revealed that differentially expressed genes (DEGs) upregulated in Day 50 (D50) old heads were enriched for immune response pathways, while those elevated in Day 10 (D10) young heads were associated with metabolic processes (Figure S1A; Table S1B). To investigate underlying epigenetic changes, native ChIP-seq for H3K4me1 and H3K27ac marks was performed on two biological replicates of D10 and D50 male head tissues. Global levels of both marks in the heads remained stable with age (Figure S1B). Principal component analysis confirmed strong reproducibility across two biological replicates (Figure S1C). H3K4me1 and H3K27ac signals were highly enriched at adult brain enhancers rather than wing disc enhancers, indicating tissue specificity (Figure S1D). High inter-sample correlations (Spearman > 0.85) further suggest that only a subset of H3K4me1 and H3K27ac peaks is altered during aging (Figure S1E).

**Figure 1:**
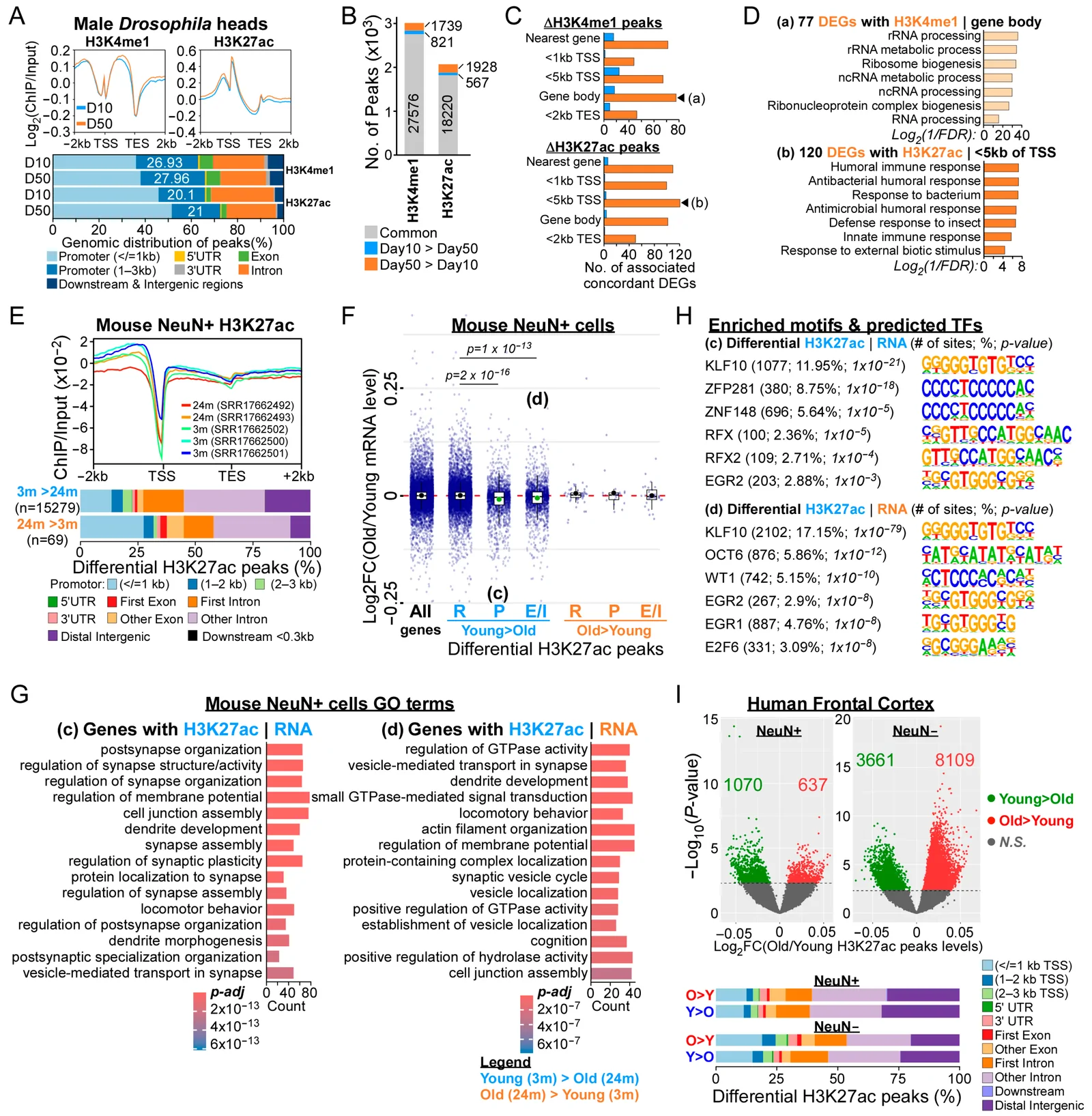
Cross-species analysis of differential H3K4me1 and H3K27ac landscapes during aging. (**A**) Metaplots of H3K4me1 and H3K27ac ChIP-seq signals across annotated genes in male *Drosophila* heads collected at Day 10 and Day 50 (top). TSS and TES indicate the transcription start site and transcription end site, respectively. The genomic distribution of H3K4me1 and H3K27ac peaks is shown below. Promoter regions are defined relative to the TSS, and UTR refers to untranslated regions. (**B**) Number of common and differential (Δ) H3K4me1/H3K27ac peaks identified between Day 10 and Day 50 male *Drosophila* heads. (**C**) Number of concordant DEGs associated with ΔH3K4me1 or ΔH3K27ac peaks across distinct genomic regions. (**D**) GO enrichment analysis of concordant DEGs associated with (a) ΔH3K4me1 peaks located within gene bodies or (b) ΔH3K27ac peaks located within 5 kb upstream of TSS. Orange indicates enrichment in Day 50 relative to Day 10 (Day 50 > Day 10). (**E**) Metaplots of H3K27ac ChIP-seq signals across annotated genes in NeuN+ neurons from young (3 months, m) and aged (24 months) mice (top). The genomic annotation of ΔH3K27ac peaks in mouse NeuN+ neurons is shown below. (**F**) Beeswarm plots showing log_2_fold change (old/young) of genes associated with ΔH3K27ac peaks located at promoters (P) or within gene bodies (exon/intron; E/I) in NeuN+ cells, compared with randomly selected gene sets (R). Statistical significance was assessed using two-sided Wilcoxon tests. (**G**) GO enrichment analysis of genes associated with elevated ΔH3K27ac peaks in young mouse NeuN+ cells that exhibit either (c) decreased or (d) increased expression during aging. (**H**) Motif enrichment analysis of elevated ΔH3K27ac peaks in young NeuN+ cells associated with genes exhibiting either decreased (c, light blue) or increased (d, orange) RNA expression during aging. Predicted transcription factor binding motifs are indicated. (**I**) Volcano plots showing age-associated ΔH3K27ac peaks (*p* < 0.005) in human NeuN+ and NeuN− cells from the frontal cortex of 29 neurologically healthy controls, with age modelled as a continuous variable (top). The genomic distribution of these ΔH3K27ac peaks is shown below.

The majority of H3K4me1 and H3K27ac peaks were distributed across promoter regions and gene bodies rather than intergenic regions, likely reflecting the relatively compact genome of *Drosophila* (Figure 1A). H3K4me1 was enriched ~1–2 kb upstream of TSSs, displaying narrow peak consistent with asymmetrical unwrapped nucleosome intermediates [16], and was also present across gene bodies. In contrast, H3K27ac peaks were localized closer to TSS, occurring ~0.5 kb upstream and ~0.2 kb downstream (Figure 1A). Analysis using MACS2 *bdgdiff* identified 821 ΔH3K4me1 peaks enriched in D10 and 1739 in D50. On the other hand, 567 ΔH3K27ac peaks were elevated in D10, compared to 1928 differential peaks enriched in D50 (Figure 1B). Overall, average peak-summit intensities for both H3K4me1 and H3K27ac differed by less than twofold between the two age groups (Figure S1F,G). Notably, there were significantly fewer concordant D10 > D50 differential peaks and DEGs compared with D50 > D10 (Figure 1C). Moreover, there was a negligible number of differential peaks associated with DEGs showing discordant expression (Table S1C). Interestingly, DEGs involved in different pathways were preferentially linked to distinct age-gained differential peaks. Specifically, ΔH3K4me1 peaks were enriched within gene bodies of DEGs involved in RNA processing and biogenesis (n = 77, panel a), whereas ΔH3K27ac peaks within 5 kb upstream of TSSs were associated with immune-related DEGs (n = 120, panel b) (Figure 1D and Table S1D). These findings suggest that H3K4me1- and H3K27ac-marked putative enhancers may be associated with distinct gene classes during aging in fly heads, while enrichment of shared and distinct DNA-binding motifs suggests potential differences in transcription factor (TF) binding preferences (Figure S1H). For example, elevated ΔH3K4me1 and ΔH3K27ac peaks in D10 heads were enriched for motifs recognized by *lethal (3) neo38*, a TF essential for neuromuscular junction formation and brain development [17]. Additionally, enrichment of *Trithorax-like* (*Trl*) binding motifs in age-gained peaks raises the possibility that *Trl* may contribute to transcriptional programs associated with aging, beyond its established role in early development [12].

### 2.2. Relative Loss and Dual Regulatory Roles of H3K27ac in Aging Mouse NeuN+ Neurons

Despite the limited number of biological replicates, ΔH3K27ac peaks in mouse NeuN+ neurons were predominantly located within gene bodies, with a substantial proportion also mapping to intergenic regions, and exhibited strong signal enrichment within 1 kb upstream of TSSs (Figure 1E). Notably, 15,279 age-associated ΔH3K27ac peaks were identified in NeuN+ neurons from 3-month-old mice (n = 3), compared with only 69 peaks in neurons from 24-month-old mice (n = 2; Table S2A–C). Genes associated with elevated promoter ΔH3K27ac peaks in young neurons showed a modest but statistically significant shift toward higher expression than matched random gene sets (Figure 1F, column c; Figure S1I). These genes were enriched for synaptic processes and dendritic development, suggesting that elevated ΔH3K27ac may be associated with transcriptional programs involved in neuronal maturation programs in young mice (Figure 1G, panel c; Table S2D). In contrast, genes involved in GTPase signalling and vesicle-related pathway genes were upregulated with age, despite reduced ΔH3K27ac levels in aged neurons (Figure 1G, panel d; Table S2E). This observation parallels previous findings suggesting that reduced H3K27ac-mediated repression may contribute to the upregulation of neuroinflammatory genes in aging mouse brains [7].

Genes associated with age-related ΔH3K27ac peaks and bidirectional expression changes were enriched for predicted EGR2 and Krüppel-like factor (KLF) family motifs, whose members regulate myelination and nervous system development, respectively (Figure 1H; Table S2F) [18,19]. In contrast, peaks associated with discordant expression changes were enriched for predicted binding motifs of transcriptional repressors, including Wilms’ tumour 1 (WT1), E2F6, and OCT6, a zinc finger TF implicated in neuronal differentiation [20].

### 2.3. ΔH3K27ac Correlates with Distinct Pathway Expression in Aging Human Brain Cells

To investigate the regulatory roles of H3K27ac in human brain aging, we identified age-associated ΔH3K27ac peaks in NeuN+ and NeuN− cells from the frontal cortex of 29 neurologically healthy controls, modelling age as a continuous variable (Table S3A). Deconvolution of bulk RNA-sequencing data from NeuN− cells revealed no significant age-related changes in the estimated proportions of five major non-neuronal cell-types (Figure S2), consistent with findings from a single-cell analysis of the aging human brain [21]. Similarly, age-stratified analyses of ChIP-seq quality metrics showed no significant age-related changes in FriP, NSC, RSC, uniquely mapped read counts, or total peak numbers in human H3K27ac datasets (Figure S3).

More age-associated ΔH3K27ac peaks were identified in NeuN− cells than NeuN+ neurons (*p* < 0.005), although their genomic distributions, as annotated using ChIPseeker, were broadly similar (Figure 1I; Table S3B,C). In NeuN+ neurons, promoter-associated ΔH3K27ac peaks showed a stronger positive correlation with age-related gene expression changes (ρ = 0.52, *p* = 0.014) than gene-body peaks (ρ = 0.17, *p* = 0.18; Figure S4A; Table S3D). In NeuN− cells, promoter- and gene body-associated peaks showed moderately strong positive correlations with age-related gene expression changes (ρ = 0.55 to 0.58, *p* < 2.2 × 10^−16^; Figure S4B; Table S3E).

**Figure 2:**
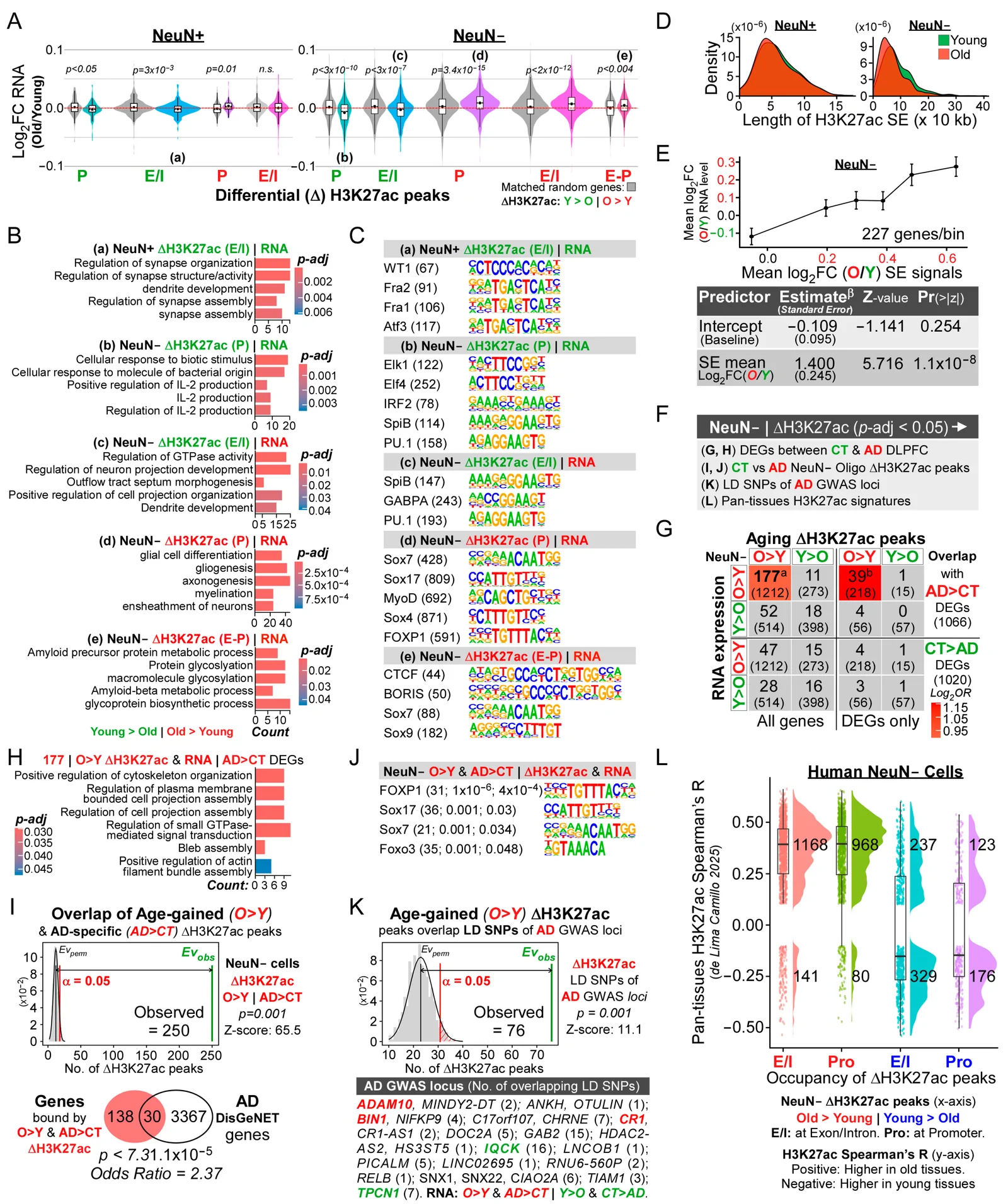
Age-dependent alterations of H3K27ac levels in human NeuN+ and NeuN− cells. (**A**) Volin plots showing log_2_fold change (old/young) in expression for genes associated with ΔH3K27ac peaks (*p* < 0.005) at promoters (P) or gene bodies (exons/introns; E/I) in NeuN+ (left) and NeuN− (right) cells, compared with matched random gene sets (grey). Parentheses (a–e) denote distinct gene groups. Statistical significance was assessed using two-tailed Wilcoxon tests. E–P denotes enhancer–promoter interactions defined by Hi-C data from NeuN− glial cells. Green and red indicate age-reduced (Young > Old) and age-gained (Old > Young) ΔH3K27ac peaks, respectively. (**B**) GO enrichment analysis of gene groups defined by age-associated changes in ΔH3K27ac and gene expression in NeuN+ and NeuN− cells. (**C**) Motif enrichment analysis of ΔH3K27ac peaks stratified by genomic location and age-associated changes in ΔH3K27ac and RNA expression in NeuN+ and NeuN− cells. (**D**) Density plots showing the size distribution of H3K27ac-marked super-enhancers (SE) in NeuN+ and NeuN− cells from young and old age groups. (**E**) Relationship between mean SE signal and RNA expression changes of associated genes, grouped into bins, in old versus young NeuN− cells (top). Logistic regression using log_2_fold changes in SE signal (O/Y) to predict the direction of RNA expression changes (bottom). O, old; Y, young. (**F**) Integrative analysis of age-related ΔH3K27ac peaks in NeuN− cells with multiple AD-associated features, including DEGs in AD dorsolateral prefrontal cortex (DLPFC), hyperacetylated ΔH3K27ac in AD NeuN− oligodendrocytes (Oligo), AD GWAS SNPs and associated linkage disequilibrium (LD) SNPs, and pan-tissue aging H3K27ac signatures. (**G**) Heatmap showing the overlap between genes associated with age-related ΔH3K27ac peaks and expression changes in NeuN− cells and DEGs in control (CT) versus AD DLPFC. Genes associated with age-gained ΔH3K27ac peaks were significantly enriched among AD > CT DEGs. OR, odds ratio. Grey boxes indicate non-significant overlap; red boxes indicate significant overlap: (**a**) adjusted *p* = 2.99 × 10^−10^; (**b**) adjusted *p* = 2.1 × 10^−4^. (**H**) GO enrichment analysis of 177 genes associated with age-gained ΔH3K27ac peaks and RNA expression changes that overlap with AD > CT DEGs. (**I**) Permutation analysis of overlap between age-gained ΔH3K27ac peaks in NeuN− cells and H3K27ac peaks with increased signal in AD NeuN− oligodendrocytes (top). The y-axis indicates the frequency distribution from permutation testing. Enrichment of genes associated with age-gained and AD-specific ΔH3K27ac peaks within the curated AD DisGeNET gene set (bottom). The *p* value was calculated by Fisher’s exact test. (**J**) Motif enrichment analysis of overlapping ΔH3K27ac peaks showing increased H3K27ac signals and concordant gene expression in aged NeuN− cells and AD NeuN− oligodendrocytes. (**K**) Permutation analysis of overlapping ΔH3K27ac peaks in NeuN− cells and AD GWAS lead SNPs and their LD proxies (top). The y-axis indicates the frequency distribution from permutation testing. The corresponding AD GWAS loci and the number of overlapping LD SNPs (in parentheses) are shown below. (**L**) Distribution of pan-tissue H3K27ac Spearman’s *R* values among genes associated with age-dependent ΔH3K27ac peaks (adjusted *p* < 0.05) in human NeuN− cells. The number of genes in each category is indicated. Red and blue denote genes associated with age-gained and age-reduced ΔH3K27ac peaks, respectively.

We next examined the relationship between the genomic locations of associated ΔH3K27ac peaks and gene expression changes. In young NeuN+ neurons, genes associated with elevated gene-body ΔH3K27ac peaks showed a modest but significant shift toward higher expression relative to randomly selected gene sets and were enriched for synaptic organization and dendritic development (Figure 2A,B and Figure S5A,B; Table S3F). HOMER motif analysis further revealed enrichment of a predicted WT1-binding motif (Figure 2C), consistent with the reported role of WT1 in regulating synaptic plasticity [22].

In young NeuN− cells, genes associated with elevated ΔH3K27ac peaks and concordant expression were enriched for cytokine production and predicted ETS-family transcription factor binding motifs (Figure 2A–C, panel b; Figure S5C,D; Table S3G,H). In aged NeuN− cells, concordantly upregulated genes were enriched for gliogenesis, myelination and amyloid-beta (Aβ) metabolic processes (Figure 2B, panels d and e). Age-gained ΔH3K27ac peaks at promoters and distal enhancers, annotated using NeuN− glial Hi-C data [23], were enriched for predicted binding motifs of SOX-family TFs and CTCF (Figure 2C; Table S3H).

### 2.4. Identification of Age-Related H3K27ac-Marked Super Enhancers in Aged NeuN− Cells

Super-enhancers (SEs) are clusters of regulatory elements associated with high expression of genes involved in cell identity and cancer [24,25]. To investigate SEs in the aging brain, we used an approach adapted from the ROSE method to identify clusters of nearby H3K27ac peaks with exceptionally high aggregate signals in the NeuN+ and NeuN− populations (Figure S6A; Table S3I). Compared with young cells, aged NeuN− cells exhibited a greater number of SEs, some of which were shorter than those observed in young cells (Figure 2D). However, further experimental validation is required to determine whether these findings reflect age-associated enhancer fragmentation, as recently reported [9]. Age-gained SEs also showed tissue-specificity, overlapping substantially with curated SEs from the nervous system but not those from other organs (Figure S6B). Although cumulative H3K27ac signals across age-related SEs differed only modestly between young and aged NeuN− cells, unlike the more pronounced changes reported in developmental and cancer contexts (Figure S6C), logistic regression revealed a positive association between age-gained SE activity and gene expression in aged NeuN− cells (Figure 2E). Despite these parallels, further profiling of additional histone modifications and TFs, coupled with perturbation experiments, will be necessary to determine whether coordinated activity of multiple regulatory elements contributes to age-related changes in gene expression.

### 2.5. Convergence of Age-Gained ΔH3K27ac Peaks in NeuN− Cells with Multimodal AD Features

Integrative analyses of healthy aging and AD brains have identified shared changes in gene expression and RNA splicing, suggesting that age-associated molecular alterations may overlap with those observed in AD [15,26,27]. To investigate the genomic convergence with AD-associated genetic and epigenetic features (Figure 2F), we focused on high-confidence age-associated ΔH3K27ac peaks in NeuN− cells that remained significant after multiple-testing correction (adjusted *p* < 0.05; Figure S7A).

Genes associated with age-dependent ΔH3K27ac peaks and expression changes in NeuN− cells were intersected with DEGs in the dorsolateral prefrontal cortex (DLPFC) of control and AD patients [28]. Gene-level overlap significance was assessed using a hypergeometric test, with the background restricted to genes evaluable in both datasets. Significant overlap was observed only between genes associated with age-gained ΔH3K27ac peaks and AD-upregulated genes, indicating a directionally concordant association between age-related H3K27ac gains and transcriptional upregulation in AD (Figure 2G; Table S3J). The 177 overlapping genes associated with age-gained ΔH3K27ac peaks and concordant expression changes were enriched for cytoskeleton organization, cell projection assembly, bleb assembly, and small GTPase-mediated signal transduction (Figure 2H).

To assess shared epigenetic changes in brain aging and AD, we examined the overlap between age-related ΔH3K27ac peaks in NeuN− cells and regions showing elevated H3K27ac in NeuN− oligodendrocyte-enriched glia from AD brains compared with healthy controls (hereafter referred to as oligodendrocytes, Oligo) [29]. Permutation analysis identified 250 overlapping sites between age-gained, but not age-reduced, ΔH3K27ac peaks and AD-associated hyperacetylated regions (Figure 2I and Figure S7B; Table S3K). In contrast, 1000 permutations using size-matched random genomic fragments yielded fewer than 15 overlaps on average, indicating that the observed overlap exceeded that expected under the random-fragment null model. Genes associated with these shared peaks, as annotated by ChIPseeker and glial Hi-C interaction data, significantly overlapped with DisGeNET-curated AD genes and were enriched for gliogenesis, oligodendrocyte development and extracellular matrix organization (Figure S7C; Table S3L). Motif analysis further revealed enrichment of predicted FOX- and SOX-family TF binding motifs (Figure 2J; Table S3M). A similar overlap was observed between age-gained, but not age-reduced, ΔH3K27ac peaks and regions with elevated H3K27ac in the lateral temporal lobes of AD brains (Figure S7D) [30]. Together, these findings suggest that some age-associated gains in H3K27ac during healthy aging are also observed in AD brains.

In the lateral temporal lobes of AD brains, disease-specific H3K27ac gains were reported to be enriched in regions containing AD-associated GWAS SNPs (*p* < 1 × 10^−5^) and their linkage disequilibrium (LD) proxies [30]. To assess whether age-associated ΔH3K27ac peaks similarly overlap AD susceptibility loci, we intersected the high-confidence peaks in NeuN− cells (adjusted *p* < 0.05) with AD-associated SNPs (*p* < 5 × 10^−8^) and their LD proxies (r^2^ ≥ 0.8; Table S3N), using 1000 permutations. Age-gained, but not age-reduced, ΔH3K27ac peaks in NeuN− cells showed significant enrichment for AD-associated variants (Figure 2K and Figure S7E; Table S3O).

Among the overlapping loci, complement receptor 1 (*CR1*) and bridging integrator 1 (*BIN1*), two prominent AD risk loci outside *APOE* [31], showed increased expression with aging and in AD. The *CR1* risk variant rs679515-T has been associated with higher expression in oligodendrocytes [32], while *CR1* expression is elevated in aging NeuN− cells and in microglia and astrocytes from AD brains [33]. *BIN1* regulates intracellular trafficking, with its roles in AD varying according to cell-type and isoform usage [34,35]. These findings highlight genomic convergence between age-associated H3K27ac gains and AD genetic risk, without establishing a causal relationship. Further studies are needed to determine whether age-related H3K27ac remodelling contributes to altered expression of these genes in AD.

### 2.6. Age-Gained ΔH3K27ac Peaks in NeuN− Cells Correlate Strongly with Pan-Tissue Signatures

Recent multivariate age-prediction analyses integrating seven histone marks across diverse human cell types identified pan-tissue age-associated changes in euchromatic marks, including H3K27ac [11]. Comparing of age-associated ΔH3K27ac profiles in NeuN− and NeuN+ cells with the pan-tissue H3K27ac signature revealed both shared and cell-type-specific patterns. Age-gained H3K27ac at promoters and gene bodies in aged NeuN− cells showed the strongest concordance with pan-tissue signature, whereas ΔH3K27ac in young NeuN− cells exhibited only modest association (adjusted *p* < 0.05; Figure 2L). In contrast, age-associated ΔH3K27ac peaks in NeuN+ cells (*p* < 0.005) showed weakest association with the pan-tissue signature (Figure S7F). These findings suggest that age-associated H3K27ac changes in frontal cortex cells encompass both shared pan-tissue signatures and cell-type-specific features.

## 3. Discussion

Comparative analyses of H3K27ac dynamics across aging *Drosophila* heads, mouse neurons, and human frontal cortex cell populations revealed both shared and cell-type-specific regulatory patterns. In aging fly heads, age-associated H3K4me1 and H3K27ac changes are linked to altered expression of distinct gene sets, resembling the distinction between developmental and housekeeping enhancers [12]. Our findings suggest that age-related transcriptomic changes in flies may reflect enhancer reconfiguration alongside de-repression associated with heterochromatin loss [36,37].

In young human and mouse NeuN+ neurons, increased H3K27ac occupancy at promoters and gene bodies was associated with higher expression of genes involved in synaptic organization and function, although the mouse findings should be interpreted cautiously given the limited number of biological replicates. Consistent with the role of enhancers in coordinating spatiotemporal gene expression, these correlations suggest that H3K27ac-marked regulatory elements may contribute to transcriptional programs supporting synaptic refinement and stabilization in early adulthood [38].

Significantly more age-associated ΔH3K27ac peaks were identified in NeuN− cells than in NeuN+ neurons. Although deconvolution analysis of bulk RNA-seq data (Figure S2) and a recent single-cell study of the aging human brain broadly found no significant changes in the overall composition of non-neuronal cell populations [21], cell-type-specific H3K27ac profiling would enable a more precise assessment of age-associated epigenetic changes within individual non-neuronal cell types.

In young human NeuN− cells, genes with altered H3K27ac occupancy and expression were enriched in pathways related to IL-2 signalling, which can modulate brain-resident regulatory T cells and neuroinflammation [39], and GTPase-mediated signalling, which regulates cytoskeletal dynamics [40]. These findings highlight potential links between chromatin remodelling, immunoregulation, and cytoskeletal organization in young non-neuronal brain cells, although pathway enrichment alone does not establish active IL-2 or GTPase signalling.

In contrast, genes upregulated in aged human NeuN− cells exhibited two distinct patterns of ΔH3K27ac occupancy. The first involved age-gained ΔH3K27ac at promoters or distal enhancers, with associated genes enriched for gliogenesis, myelination and Aβ metabolic processes (Figure 2B). These peaks were enriched for predicted binding motifs of SOX-family TFs and CTCF. Notably, SOX9 has been implicated in maintaining astrocyte function in aging hippocampus and promoting Aβ plaque phagocytosis in an AD mouse model [41]. CTCF, a nuclear architectural protein, mediates long-range chromatin interactions [42]. Consistent with potential role of chromatin architecture in brain aging, recent single-cell three-dimensional (3D) genome mapping of aging human hippocampus revealed age-associated changes in genome organization alongside cell-type-specific epigenetic and transcriptional alterations [43]. Further investigation of SOX9- and CTCF-associated regulatory networks in aging non-neuronal populations and AD brain may provide mechanistic insight into the transcriptional changes accompanying the transition from healthy aging to AD pathogenesis. The second pattern was characterized by reduced ΔH3K27ac occupancy across gene bodies (Figure 2B, panel c). Further perturbation studies are needed to determine this reduction contributes to gene de-repression, as reported for specific neuroinflammatory genes [7].

Epigenomic profiling of AD brains has identified H3K27ac enrichment at AD-associated genes, including *APP*, *PSEN1/2*, *BIN1*, and *PICALM* [29,30,44]. Our analyses revealed three levels of convergence between age-gained ΔH3K27ac peaks in NeuN− cells and AD-associated features: (1) gene-level overlap with DEGs upregulated in the AD DLPFC; (2) genomic-region overlap with AD-specific ΔH3K27ac gains in oligodendrocytes and the lateral temporal lobes, including regions associated with DisGeNET-curated AD genes; and (3) enrichment of AD GWAS loci and their LD proxies, including *BIN1* and *CR1*, within age-gained peaks. Together, these findings highlight shared genetic and epigenomic associations between healthy aging in non-neuronal frontal cortex cells and AD.

## 4. Materials and Methods

### 4.1. Fly Strains and Husbandry

Male *w^1118^ Drosophila melanogaster* was cultured in standard fly media (6% cornmeal, 5% dextrose, 2.4% brewer’s yeast, 0.8% agarose, and 0.3% nipagin). Twenty male flies were housed in a cylindrical vial (height 9.5 cm, diameter 2.5 cm) at 25 °C with 12-h day/night cycle. Eclosed adults were considered as day 0 and vial was changed every two days until Day 10 or Day 50. Flies were frozen in liquid nitrogen and stored at −80 °C until enough animals were collected for downstream experiment.

### 4.2. Immunoblot

Fly heads dissected from flies under light CO_2_ anaesthesia were homogenized with plastic pestle (Sigma, St Louis, MO, USA, Cat#Z359947) in 100 μL of 1× Laemmli buffer (62.5 mM Tris-HCl pH 6.8, 2% SDS, 8.3% glycerol, 0.01% bromophenol blue, 0.1 M DTT). After boiling, lysate was resolved on SDS-polyacrylamide gel and transferred to PVDF membrane using standard protocol. After blocking with 5% milk in TBST (0.05% Tween-20), the membranes were probed with the H3K4me1, H3K27ac (see below, 1:5000) and β-tubulin (1:2000, DSHB, Iowa City, IA, USA, Cat#E7-s) antibodies. Following washes and secondary antibodies staining, immunoblots were developed with SuperSignal™ West Pico PLUS chemiluminescent substrate (Thermo Scientific, Waltham, MA, USA, Cat# 34578).

### 4.3. Native MNase Chromatin Immunoprecipitation (ChIP) and Sequencing

Two biological replicates were performed for each experiment, with 50 mg of fly heads collected at either Day 10 or Day 50 for each replicate. Fly heads were dounced 30 times with pestle A (Sigma) in 5 mL of A1 buffer (60 mM KCl, 15 mM NaCl, 4 mM MgCl_2_, 15 mM HEPES pH 7.6, 0.5% NP-40, 0.5 mM DTT, 0.5 mM PMSF, and protease inhibitors cocktail). Samples were filtered with MIRA cloth to obtain lysates. Following 15 min of incubation on ice, lysates were centrifuged at 4000× *g* for 5 min at 4 °C. After three additional rounds of A1 buffer wash and centrifugation, nuclear pellet was resuspended and dounced with pestle B (Sigma) in 3.3 mL of RIPA buffer (10 mM Tris pH 8.0, 1 mM EDTA, 140 mM NaCl, 1% Triton, 0.1% SDS, 0.1% sodium deoxycholate, 0.5 mM DTT, 0.5 mM PMSF, and protease inhibitors cocktail). Then, 3.3 µL of 3M CaCl_2_ was added to 3.3 mL of nuclear lysate and incubated at 37 °C for 3min; 5 µl of MNase (i-DNA Biotech, Singapore, Cat# LS004798) was then added to samples and incubated at 37 °C for 15 min. MNase reaction was terminated by adding STOP buffer (5 µM EDTA/EGTA). Samples were briefly sonicated (10 s ON, 50 s OFF, 5 times), followed by 10 min max speed centrifugation at 4 °C. The supernatant was collected as the chromatin extract and 80 µL was kept as input. Then, 5 µL of H3K4me1 (Abcam, Cambridge, UK, ab8895) or H3K27ac (Abcam, ab4729) antibody was added to remaining chromatin extract and incubate in 4 °C overnight. On the following day, 20 μL of pre-blocked (0.5% BSA in PBS) Protein-G Dynabeads™ (Lifetech, Waltham, MA, USA, Cat# 10003D) was added to the chromatin extract. Following 4 h of incubation at 4 °C, DNA-antibody-beads complex was isolated using magnetic stand. The beads complex was washed with RIPA buffer 5 times, LiCl buffer (10 mM Tris pH 8, 1 mM EDTA, 250 mM LiCl, 0.5% NP-40, 0.5% sodium deoxycholate) once, and Tris-EDTA buffer (10 mM Tris pH 8, 1 mM EDTA) once. The DNA fragments were eluted from the beads with 500 μL elution buffer (1 M NaHCO_3_, 0.5% SDS), while input was topped up to 420 µL with elution buffer and samples were incubated at 37 °C overnight. On the following day, samples were treated with RNase A (20 μg/ml, 1 h at 37 °C) and proteinase K (40 μg/ml, 2 h at 37 °C). DNA was extracted by phenol/chloroform and ethanol precipitation. Barcoded libraries were generated for H3K4me1 and H3K27ac ChIP DNA by KAPA real-time library amplification kit (KAPA Biosystems, Darmstadt, Germany) according to the manufacturer’s instructions and sequenced on Illumina Hiseq 4000 SE 50 at BGI Tech Solutions (Shenzhen, China).

### 4.4. Male Fly Head RNA-Seq Datasets Used

RNA-sequencing (RNA-seq) data from male head tissues of *w^1118^ Drosophila melanogaster* collected at days 10, 20, 30, and 50 (D10, D20, D30, and D50), with three biological replicates per age group, were previously reported (GSE110349) [15].

### 4.5. Male Fly Heads RNA-Seq Analysis

Mapped RNA-seq reads from fly heads [15] were counted with HTSeq-count (v.0.7.2) [45]. DESeq2 [46] was then used to compute differential gene expressions between flies at different ages.

### 4.6. Analysis of Male Fly Heads ChIP-Seq Data

FastQC (https://www.bioinformatics.babraham.ac.uk/projects/fastqc/ accessed on 7 February 2022) was used to assess quality of ChIP-seq data and the sequencing reads were mapped to *Drosophila* genome (BDGP6.22.98) using STAR (v2.5.3a) [47]. Two independent biological replicates were analysed for each age group.

H3K27ac and H3K4me1 peaks were called using MACS2 [48] with the following parameters: --no model --extsize 147 –bdg. For each age group, the two biological replicates were pooled and jointly analysed using MACS2 callpeak. Differential histone modifications peaks were identified using MACS2 bdgdiff function with cut-off at one. ChIPseeker [49] was used to annotate the genomic features of the differential histone modifications peaks, including promoter, UTR, exon, intron, downstream, and intergenic regions.

The deepTools2 suite [50] was used for downstream analyses. Read counts from BAM files at the peak regions were obtained using *multiBamSummary* and used for principal component analysis (PCA). *BamCompare* was used to generate BPM-normalised BigWig files from Input and ChIP BAM files. BigWig scores were calculated with *multiBigWigSummary* and used to compute Spearman correlation coefficients between different samples, with resulting heatmap generated using the *plotCorrelation* tool.

Quantitative ChIP-seq signal was used to generate metaplots. Depth-normalised ChIP and input BAM files were merged across biological replicates, and *bamCompare* was used to generate BPM-normalised BigWig files representing log_2_(ChIP/input) signal. Metaplots were then generated from these BigWig files using *computeMatrix* and *plotHeatmap*.

### 4.7. Assignment of Differential H3K4me1 and H3K27ac Peaks to DEGs

Differential H3K4me1 and H3K27ac peaks were assigned to DEGs using the same strategies for determining enhancer-to-gene interactions as described in fly [12]: (1) ‘Nearest gene’, where each differential peak is assigned to the closest gene (only one gene per peak); (2) ‘<1 kb TSS’, where peaks are assigned to all DEGs with TSSs within 1 kb; (3) ‘<5 kb TSS’, where the peak is within 5 kb upstream of the TSSs of DEGs; (4) ‘Gene body’, where peaks are located within a DEG’s gene body (multiple genes allowed); and (5) ‘2 kb TES’, where peaks are within 2 kb downstream of the DEG’s TES (multiple genes allowed).

### 4.8. Human Datasets Used

Publicly available H3K27ac MOWChIP-seq and RNA-seq datasets from NeuN+ and NeuN− nuclei isolated from post-mortem human frontal cortex (Brodmann area 9) were obtained from PsychENCODE [10]. Age-associated analyses were restricted to neurological healthy control subjects (n = 29). Subject metadata, including drugs detected in blood at the time of death, are provided in Table S3A. Samples were stratified into three age groups: young (≤30 years, n = 7), middle-aged (31–50 years, n = 14), and old (>50 years, n = 8).

Published AD-associated H3K27ac datasets from oligodendrocyte-enriched NeuN− cells [29] and lateral temporal lobes [30], promoter-enhancer interaction maps from NeuN+ and NeuN− cells [23], and single-cell RNA-sequencing datasets from control and AD prefrontal cortex [51,52] were downloaded and integrated for analysis.

### 4.9. Differential H3K27ac Peak Analysis of Aging NeuN+ and NeuN− Cells

Reads were aligned to the human genome (hg38, Ensembl v107) using STAR (v2.7.10a). ChIP-seq peaks were called using MACS2 [48] without an input control, as the only available input sample, derived from a young individual, was not an appropriate matched control for all the 29 ChIP-seq samples spanning different age groups. The absence of matched input controls is acknowledged as a limitation of this study.

Instead, H3K27ac peaks were identified based on ChIP signal enrichment, with replicate consistency used for downstream filtering. Peaks were processed using DiffBind package [53] (http://bioconductor.org/packages/release/bioc/html/DiffBind.html accessed on 20 June 2025).

Replicate-level consensus peaks (consensus = DBA_REPLICATE, minOverlap = 2) were generated and merged into a unified peak set, restricting analyses to reproducible regions across replicates. Read counts for each sample were then quantified over this consensus peak set for downstream analyses.

Differential analysis of H3K27ac peaks was performed using the limma–voom framework [54,55]. Low-signal peaks were filtered using *filterByExpr* to remove regions with insufficient coverage. Library sizes were normalized using the trimmed mean of M-values (TMM) method. The linear model incorporated sex, additional drug exposure, post-mortem interval (PMI), percentage of uniquely mapped reads, fraction of reads in peaks (FRiP), normalized strand cross-correlation (NSC), and relative strand cross-correlation (RSC) as covariates. Two technical replicates per sample were accounted for using *duplicateCorrelation* to model within-sample correlation. Voom transformation, linear modelling, and empirical Bayes moderation were then applied. Age was modelled as a continuous variable to identify age-associated changes in H3K27ac signal. ΔH3K27ac peaks with nominal *p* < 0.005 were used to characterize age-associated epigenetic changes and their relationships with gene expression in NeuN+ and NeuN− cells. For downstream analyses investigating the convergence of age-associated changes with AD-related genetic and epigenetic features, a more stringent threshold of multiple-testing-adjusted *p* < 0.05 was applied to ΔH3K27ac peaks in NeuN− cells.

### 4.10. Differential Gene Expression Analysis

Gene-level read counts from NeuN+ and NeuN− RNA-seq datasets (GSE174407) [10] were analysed using the limma–voom pipeline. The design matrix incorporated biological and technical covariates to control for potential confounders, including sex, PMI, additional drug exposure, uniquely mapped rate, duplication rate, exon mapping rate, and GC content.

Lowly expressed genes were filtered using filterByExpr, retaining genes with ≥10 counts in at least half of the samples. Library size normalization was performed with the TMM method (calcNormFactors). To account for correlations between technical replicates or samples from the same individual, within-block correlations were estimated using duplicateCorrelation. Gene counts were then transformed with voom, followed by linear modelling (lmFit) and empirical Bayes moderation (eBayes).

### 4.11. Cell-Type Deconvolution of Bulk RNA-Seq Data from NeuN− Populations

Cell-type proportions were estimated for each bulk RNA-seq library using BisqueRNA with the ReferenceBasedDecomposition function [56], using annotated cell types from a human DLPFC single-nucleus RNA-seq dataset as reference profiles [57]. Marker genes were selected using the mean expression ratio approach implemented in DeconvoBuddies (DOI: 10.18129/B9.bioc.DeconvoBuddies), retaining the top 25 ranked genes per cell type. Only genes shared between the reference and bulk RNA-seq datasets were included in the analysis. Associations between age and estimated cell-type proportions were assessed separately at the technical replicate and donor levels using Spearman’s rank correlation. For donor-level analyses, proportions were averaged across the two technical replicates per donor. *p* values were adjusted for multiple testing using the Benjamini–Hochberg method.

### 4.12. ChIP-Seq Peak Annotation and Functional Enrichment

Differential H3K27ac peaks identified between conditions were annotated to genomic features using ChIPseeker. Peaks were assigned to promoters (±3 kb from TSS), introns, exons, and intergenic regions based on the GRCh38/hg38 gene annotation. Each peak was linked to the corresponding gene to generate a peak-associated gene list for downstream analyses.

Functional enrichment analyses were performed on these peak-associated genes to assess their biological significance. Gene Ontology (GO) biological process (BP) enrichment was assessed using clusterProfiler [58], and pathway-level enrichment was evaluated using ReactomePA [59]. Enriched terms with Benjamini–Hochberg adjusted *p* < 0.05 were considered statistically significant.

### 4.13. Identification of Age-Associated H3K27ac-Marked Super-Enhancers (SEs)

For identification of age-associated super-enhancers (SEs), subjects were stratified into young (≤30 years; n = 7) and old (>50 years; n = 8) groups. Consensus peaks in NeuN+ and NeuN− nuclei were stitched following the ROSE workflow [24,60]. Peaks overlapping promoters (±2 kb from TSS) were excluded, and the remaining peaks within 12.5 kb of each other were stitched into enhancer regions. Read counts for each stitched enhancer were calculated by summing counts across its constituent H3K27ac peaks, followed by TMM normalization and conversion to counts per million (CPM).

Within each age group (young vs. old), stitched enhancers were ranked by H3K27ac signal intensity, and the inflection point of the ranked signal curve was used to distinguish SEs from typical enhancers. A union set of SEs identified across both age groups was generated for downstream analyses. Differential H3K27ac signal across stitched enhancers was assessed using limma–voom, adjusting for donor, technical, and biological covariates. SEs with an adjusted *p* < 0.05 were considered significantly different between age groups.

### 4.14. Gene Assignment of Differential H3K27ac Peaks

Differential H3K27ac peaks identified at *p* < 0.005 and adjusted *p* < 0.05 were annotated to genomic features using ChIPseeker. Promoter regions were defined as ±3 kb from the TSS, while intragenic regions comprised exons and introns. Promoter peaks were assigned to the nearest TSS-associated gene, and intragenic peaks were assigned to the gene containing the peak, based on ChIPseeker annotations. Distal peaks were assigned to genes using glial Hi-C interaction data [23].

### 4.15. Correlation of Differential H3K27ac Peaks with Gene Expression

The relationship between age-associated changes in ΔH3K27ac peaks (*p* < 0.005) and gene expression was investigated by assigning each annotated peak to its corresponding gene and integrating these annotations with age-associated RNA-seq expression changes from matched datasets. For each peak category, the distribution of age-associated RNA log_2_ fold changes was compared with those of five independently sampled random gene sets of equal size from the RNA-seq gene pool. Differences were assessed using the Wilcoxon rank-sum test, and distributions were visualized using violin plots or jittered boxplots. To further assess the robustness of these comparisons, 1000 random gene sets of equal size were independently sampled from the RNA-seq gene pool. The median RNA log_2_ fold change was calculated for each random gene set to generate an empirical null distribution, against which the observed median was compared.

Expression of genes associated with H3K27ac peaks in NeuN−, oligodendrocyte-enriched glia [29] was assessed using single-cell RNA-seq data from control and AD prefrontal cortex samples [52].

### 4.16. Integrative Analyses of Multi-Omic Human Datasets

Genomic coordinates of AD-specific ΔH3K27ac peaks and enhancer-promoter interaction maps were converted from hg19 to hg38 using the UCSC liftOver tool [61]. Significance of genomic overlap between datasets was assessed by permutation testing (1000 permutations) using the regioneR R package [62]. Motif enrichment analysis was performed using HOMER [63]. Enrichment for overlap with curated AD-associated genes from DisGeNET [64] was evaluated using Fisher’s exact test implemented in *GeneOverlap* [65]. Gene ontology (GO) biological process enrichment analysis was performed using clusterProfiler [66].

Disease-related DEGs (CT > AD and AD > CT) were defined for AD DLPFC using an adjusted *p* < 0.05 [28]. Age-related ΔH3K27ac peaks were assigned to genes based on promoter, intragenic and distal intergenic annotations, as described above. Gene set overlap analyses were then performed to assess the relationship between AD-associated DEGs and genes exhibiting age-related changes in H3K27ac and RNA expression. Overlap significance was evaluated using a hypergeometric test implemented in *GeneOverlap* [65], using genes testable in both the aging and AD datasets as the background gene universe. Results were summarised in a matrix according to the direction of AD-associated RNA changes and age-related changes in RNA expression and H3K27ac. Heatmaps show the corresponding log_2_ odds ratios, with overlap counts and the sizes of the respective gene sets indicated.

Published AD-specific ΔH3K27ac peaks from purified NeuN−, oligodendrocyte-enriched glia [29], and lateral temporal lobe [30] were intersected with age-associated ΔH3K27ac peaks in NeuN− cells using a minimal overlap of 1 bp. AD-associated variants were obtained from the GWAS Catalog [67], using a genome-wide significance threshold of *p* < 5 × 10^−8^. LD proxies were identified using LDlinkR [68], selecting the 1000 Genomes reference population to match the ancestry of the study reporting each lead SNP and retaining variants with r^2^ ≥ 0.8. For each lead SNP, the number of identified LD proxies and the genomic region spanning the lead SNP and its proxies were recorded (Table S3N). This approach has previously been used to examine the overlap between neuronal enhancers with AD-associated variants and their LD proxies [69].

Lead SNPs and their LD proxies were then intersected with age-associated ΔH3K27ac peaks in NeuN− cells (adjusted *p* < 0.05). Statistical significance of genomic overlap was assessed using regioneR with 1000 permutations. For analyses comparing age-associated peaks with published AD-associated H3K27ac peaks, age-associated H3K27ac regions were randomly relocated using randomizeRegions, preserving their original region sizes. For analyses involving AD-associated lead SNPs and LD proxies, the variant positions were randomised to generate null distribution.

### 4.17. Analysis of Age-Related H3K27ac Changes in Mouse Neurons

To evaluate age-associated H3K27ac changes in mouse brain, we downloaded H3K27ac ChIP-seq datasets from forebrain NeuN+ neurons, comprising young (3 months, n = 3) and aged (24 months, n = 2) mice [6]. Differential binding between age groups was analysed using DiffBind with dba.contrast() and dba.analyze(). Differentially bound peaks were defined using an FDR-adjusted *p* < 0.05. Differential peaks were annotated to their associated genes using ChIPseeker. H3K27ac changes were then integrated with RNA expression profiles from Ximerakis et al. (2019) [70]. Correlation distributions were visualized using the same approach applied to human samples.

### 4.18. Motif Analysis for Drosophila ChIP-Seq Data

Motif enrichment was performed using MEME-ChIP on the sequences of differential peaks identified in male *Drosophila* heads [71]. Analyses were run in DNA mode with a standard background model, searching for motifs ~5–30 bp in length. MEME, STREME, and CentriMo were executed with default settings [72].

### 4.19. Linking SEs to Age-Related Gene Expression Changes

Age-related SEs identified in human NeuN− cells were assessed for tissue specificity and their association with RNA expression directionality. SE regions were intersected with annotated SEs from the dbSUPER database [73]. Enrichment of SE overlap across tissue groups was evaluated using Fisher’s exact tests based on 2 × 2 contingency tables comparing overlapping and non-overlapping SEs. Results were visualised as bubble plots of log_2_(odds ratio) versus −log_10_(*p*-value).

RNA expression directionality as a function of SE activity during aging was assessed by first defining SE-gene associations based on genomic overlap with genes. Gene-level SE activity was then calculated as the average log_2_ fold-changes in H3K27ac signals across all SEs assigned to each gene. The association between SE activity and gene expression directionality, based on RNA-seq data, was tested using logistic regression, with mean SE activity as the predictor and the binary gene expression change (age-up or age-down) as the outcome.

For visualization, genes were stratified into six quantiles based on mean SE activity. Mean age-related RNA expression changes were computed within each bin to illustrate the relationship between SE strength and transcriptional output.

### 4.20. Evaluation of Differential H3K27ac Peaks with Pan-Tissue Histone Aging Predictor

To assess the broad applicability of the pan-tissue histone age predictor, age-associated ΔH3K27ac peaks identified in NeuN+ and NeuN− cells (adjusted *p* < 0.05 for Figure 2L and *p* < 0.005 for Figure S7F) were compared with the published pan-tissue H3K27ac Spearman’s R coefficients [11]. Each gene associated with a ΔH3K27ac peak was mapped to the published pan-tissue H3K27ac atlas, which reports Spearman correlation coefficients between H3K27ac levels and age across multiple tissues. Correlation distributions were examined by peak location (promoter versus gene body) and direction of age-associated change (Old > Young versus Young > Old). Distributions were visualized using raincloud plots comprising of half-violins, boxplots, and jittered individual data points.

## Data Availability

Raw and processed H3K4me1 and H3K27ac ChIP-seq data from aging male *Drosophila* are available under accession number GSE210427.

## Supplementary Materials

The following supporting information can be downloaded at: https://www.mdpi.com/article/10.3390/cells15212007/s1, Figure S1. Characterization of differential H3K4me1 and H3K27ac peaks in Drosophila. (A) Heatmap and GO terms of differentially expressed genes (DEGs) in Day 10 (D10) and Day 50 (D50) male fly heads. (B) Immunoblot analysis showing global levels of H3K4me1 and H3K27ac in whole-head lysates from male flies collected at the indicated time-points. β-tubulin serves as loading control. (C) Principal component analysis (PCA) of ChIP and input samples from Day 10 and Day 50 male fly heads. PCA was performed using genome-wide signal profiles for H3K4me1 and H3K27ac along with their corresponding inputs. (D) H3K4me1 and H3K27ac enrichment at tissue-specific enhancers. Metaplots depict signal profiles on Day 10 and Day 50 over adult brain enhancers (left) and wing disc enhancers (right). (E) Spearman correlation heatmaps of H3K4me1 (left) and H3K27ac (right) ChIP-seq samples from two biological replicates of male Drosophila heads collected at Day 10 and Day 50. (F) Metaplots showing the distribution of H3K4me1 and H3K27ac ChIP-seq signals across differentially expressed genes (DEGs) in male Drosophila heads collected at Day 10 and Day 50. TSS and TES denote the transcription start site and transcription end site, respectively. (G) Metaplots of H3K4me1 and H3K27ac ChIP-seq signals centered on the summits of common and age-differential H3K4me1/H3K27ac peaks identified between Day 10 and Day 50 in male Drosophila heads. (H) Motif enrichment analysis of age-dependent ΔH3K4me1 and ΔH3K27ac peaks, with the predicted transcription factor (TF) binding to each motif indicated. (I) Beeswarm boxplots illustrating RNA log_2_ fold changes of genes associated with young > old ΔH3K27ac peaks in mouse forebrain NeuN+ neurons, compared with five size-matched random gene sets (R). “P” and “E/I” denote promoter and exon-intron peak locations, respectively. Figure S2. Cell-type deconvolution of human NeuN− cells across donor age. (A) Stacked bar plots showing the estimated proportions of astrocytes (salmon), endothelial/mural cells (olive), microglia (green), oligodendrocytes (blue), and oligodendrocytes precursor cells (OPC; magenta) in human NeuN− cell populations, estimated by deconvolution of bulk RNA-seq data. Each bar represents one technical replicate from an individual donor. Donor IDs, age at death, and sex are indicated below each bar; two technical replicates were analysed per individual. (B) Associations between estimated cell-type proportions and donor age. Each point represents an individual technical replicate (top) or the mean of two technical replicates per donor (bottom). Lines indicate fitted linear regressions and 95% confidence bands. Spearman correlation coefficients (ρ) and adjusted *p*-values are shown in each panel. Age is presented in years. Figure S3. Association between donor age and ChIP-seq quality control metrics. Individual ChIP-seq quality control metrics from (A) NeuN+ and (B) NeuN− cells plotted against donor age. Only percent of uniquely mapped reads in NeuN+ neurons is modestly correlated with age of individuals. Figure S4. Effects of age-related changes in differential H3K27ac signals on the RNA expression of the associated genes in human NeuN+ and NeuN− cells. (A–B) Scatterplots showing the relationship between RNA log_2_ fold change (old/young; y-axis) and ΔH3K27ac signal (Δ peaks with *p* < 0.005; x-axis) in aging human (A) NeuN+ and (B) NeuN- cells. Left, genes associated with ΔH3K27ac peaks at promoters; right, genes associated with ΔH3K27ac peaks located in exon-intron regions. Red and green points indicate DEGs (*p* < 0.05), while grey points represent non-DEGs. Genes associated with ΔH3K27ac peaks without detectable RNA expression were excluded. The dashed dark red line indicates linear regression fit, and the grey region represents the corresponding confidence interval. Figure S5A–B. Statistical comparison of expression patterns of genes associated with ΔH3K27ac peaks and matched random genes in human NeuN+ cells. (A) Histograms showing the distributions of median RNA log_2_ fold-change values for 1000 randomly sampled gene sets, each matched in size to the set of genes associated with ΔH3K27ac peaks at distinct genomic loci in NeuN+ neurons. The reported z and *p* values indicate the standardized deviation of the observed median from the permutation distribution and its statistical significance, respectively. Red dashed lines indicate the observed median RNA log2FC for each gene set. (B) Beeswarm boxplots showing RNA log_2_ fold changes for genes associated with (left) young > old and (right) old > young ΔH3K27ac peaks, compared with an equal number of randomly selected genes in NeuN+ cells from human frontal cortex. Genes were grouped according to the genomic location of the ΔH3K27ac peaks at promoters, exon-intron regions and distal enhancers inferred from neuronal Hi-C data. Figure S5C–D. Statistical comparison of expression patterns of genes associated with ΔH3K27ac peaks and matched random genes in human NeuN− cells. (C) Histograms showing the distributions of median RNA log_2_ fold-change values for 1000 randomly sampled gene sets, each matched in size to the set of genes associated with ΔH3K27ac peaks at distinct genomic loci in NeuN− cells. The reported z and *p* values indicate the standardized deviation of the observed median from the permutation distribution and its statistical significance, respectively. Red dashed lines indicate the observed median RNA log_2_FC for each gene set. (D) Beeswarm boxplots showing RNA log_2_ fold changes for genes associated with (left) young > old and (right) old > young ΔH3K27ac peaks, compared with an equal number of randomly selected genes in NeuN− cells from human frontal cortex. Genes were grouped according to the genomic location of the ΔH3K27ac peaks at promoters, exon-intron regions and distal enhancers inferred from glial Hi-C data. Figure S6. Age-dependent differential super-enhancers (SE) in NeuN− cells. (A) Definition of H3K27ac-marked super-enhancers “SEs” in NeuN+ (top) and NeuN− (bottom) cells using the ROSE approach. Individual H3K27ac peaks were stitched into broader enhancer regions based on genomic proximity and ranked by normalized H3K27ac signal intensity. SEs are defined as enhancer regions above the inflection point of the curve, indicated by the red vertical dashed line. (B) Overlap analysis of 1303 SEs identified in aged NeuN− cells with tissue-specific super-enhancers curated from the dbSUPER database revealed strong cell-type specificity. (C) Beeswarm plot showing stitched H3K27ac regions and super-enhancers (SEs) in NeuN− cells from young and aged individuals. Differential SEs (ΔSE) are connected by grey lines. H3K27ac signal intensity for each stitched region was normalized as counts per million (CPM). Figure S7. Multimodal analysis of ΔH3K27ac identified in NeuN− cells. (A) Volcano plots showing ΔH3K27ac peaks (p-adjusted < 0.05) identified in NeuN+ neurons (left) and NeuN− cells (right) from human frontal cortex using a continuous age model. Only age-related ΔH3K27ac peaks from NeuN− cells were used for comparative analysis with AD datasets in Figure 2F onward. (B) Permutation analysis of overlap between age-reduced ΔH3K27ac peaks in NeuN− cells and hyperacetylated H3K27ac peaks in AD NeuN− oligodendrocytes. Y-axis indicates the frequency distribution from 1000 permutations. Related to Figure 2I. (C) (Top) List of curated AD genes from DisGeNET database associated with AD-specific and age-up ΔH3K27ac peaks in NeuN− cells. (Bottom) GO terms enrichment of genes bound by AD-specific and age-up ΔH3K27ac peaks in NeuN− cells. Related to Figure 2I. (D) Permutation analysis of the overlap between age-related ΔH3K27ac peaks in NeuN− cells and ΔH3K27ac peaks identified in control versus AD lateral temporal lobe (LTL) samples. The y-axis indicates the frequency distribution of observing the overlap generated by permutation testing. (E) Permutation analysis of the overlap between age-down (Y>O) ΔH3K27ac peaks in NeuN− cells and SNPs in linkage disequilibrium (LD) with AD GWAS lead variants. The y-axis represents the frequency distribution of overlap from 1000 permutations. Related to Figure 2K. (F) Distribution of pan-tissue H3K27ac Spearman’s R values for genes associated with age-dependent ΔH3K27ac peaks (*p* < 0.005) in human NeuN+ (top) and NeuN− (bottom) cells. Numbers indicate genes in each category. Red and blue denote age-up and age-down ΔH3K27ac peaks in frontal cortex cells, respectively (x-axis). Positive and negative R values indicate higher H3K27ac levels in old and young pan-tissue samples, respectively (y-axis). Related to Figure 2L. Table S1: Drosophila datasets. (A) Differential expressed genes (DEGs) between D10 and 50 male fly heads. (B) GO terms of DEG with statistically significant differences in their expression between Day 10 and 50 male fly heads. (C) Number of DEGs assigned to concordant or discordant differential peaks. (D) GO terms of DEGs associated with either (i) differential H3K27ac peaks less than −5kb of their TSS (D50 > D10) or (ii) H3K4me1 peaks in gene body (D50 > D10). Table S2: Mouse datasets. (A) Metadata of H3K27ac ChIP-seq of NeuN+ neurons from 3- & 24-months old mice. (B) Differential H3K27ac peaks (3 > 24 months) in mouse NeuN+ cells. (C) Differential H3K27ac peaks (24 > 3 months) in mouse NeuN+ cells. (D) GO terms of genes with differential H3K27ac at promoter (Y>O) & aged-dependent alterations in mRNA expression (Y>O or O>Y). (E) GO terms of genes with differential H3K27ac in exon-intron (Y>O) & aged-dependent alterations in mRNA expression (Y>O or O>Y). (F) Enriched motifs and predicted transcription factors at ΔH3K27ac peaks (Y>O) associated with concordant and discordant mRNA expression (Y>O or O>Y). Table S3: Human datasets. (A) Metadata & QC metrics of neurological healthy controls from (phs002487.v1.p1). (B) Age-gained and age-reduced differential H3K27ac peaks in NeuN+ cells. (C) Age-gained and age-reduced differential H3K27ac peaks in NeuN− cells. (D) List of age-associated differential expressed genes (DEGs) in NeuN+ cells. (E) List of age-associated differential expressed genes (DEGs) in NeuN− cells. (F) Top 20 enriched GO terms of genes associated with Y>O differential H3K27ac peaks at exon/intron and Y>O expression in NeuN+ cells. (G) Enriched GO terms of genes associated with age-related differential H3K27ac peaks and expression changes in NeuN− cells. (H) Enriched motifs and their predicted transcription factors (TFs) at various differential H3K27ac peaks in NeuN+/−cells. (I) List of super enhancers defined by stitched H3K27ac peaks in different age groups of NeuN− and NeuN+ cells with their respective CPM scores. (J) GO terms of protein-coding genes with age-related ΔH3K27ac peaks and expression changes overlapping with AD DEGs. (K) Overlap of ΔH3K27ac peaks elevated in aged NeuN− cells (age-gained) and AD NeuN− oligodendrocytes (AD-specific). (L) GO terms of genes associated with both O>Y & AD > CT ΔH3K27ac peaks in NeuN− cells. (M) Enriched motifs & predicted TFs for overlapping age-gained and AD-specific ΔH3K27ac peaks with concordant gene expression. (N) Summary of AD GWAS lead SNPs and the number of LD proxies. (O) Overlap of differential H3K27ac peaks (O>Y) in NeuN− cells with LD SNPs of AD GWAS loci.
