# Supplementary material for: Cross-Species and Cell-Type-Specific H3K27ac Dynamics Link Brain Aging to Alzheimer’s Disease Genes
Source: Cells. 2026 Nov 4;15(21):2007. doi: 10.3390/cells15212007 (PMC13761543; doi:10.3390/cells15212007)
Supplement: Supplementary file 1 [file cells-15-02007-s001.zip › cells-4546691-supplementary.pdf]

SUPPLEMENTAL INFORMATION (SI)

CONTENT

1. Supplemental Figures S1 to S7 and their legends.
2. Description of Supplemental Table S1 to S3.

https://drive.google.com/drive/folders/1kr5Xdn3TI4UBYVQ09f9bTafecr6WifHv

1 | SUPPLEMENTAL FIGURES

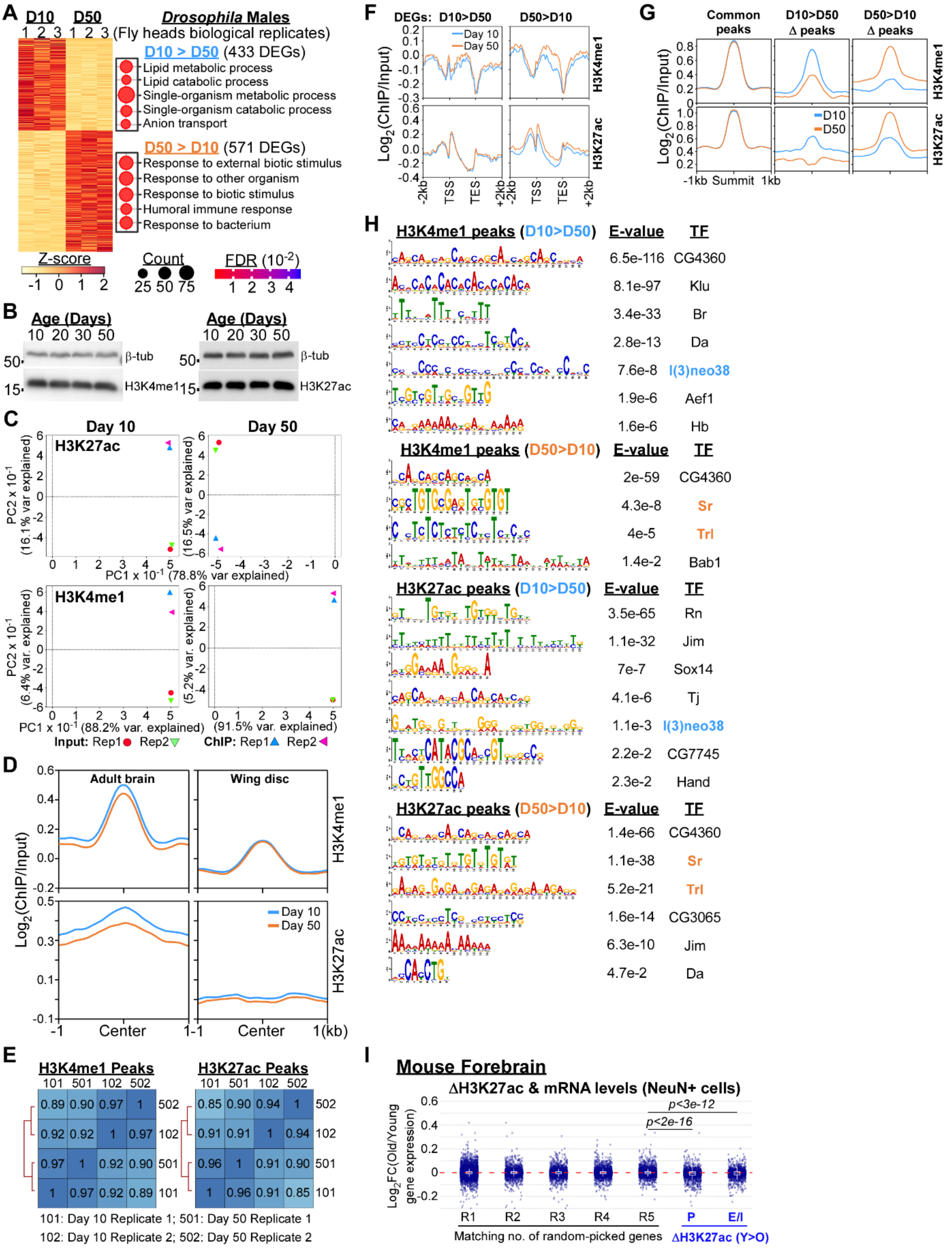

Figure S1. Characterization of differential H3K4me1 and H3K27ac peaks in *Drosophila*. (A) Heatmap and GO terms of differentially expressed genes (DEGs) in Day 10 (D10) and Day 50 (D50) male fly heads.

(B) Immunoblot analysis showing global levels of H3K4me1 and H3K27ac in whole-head lysates from male flies collected at the indicated time-points. β-tubulin serves as loading control.

(C) Principal component analysis (PCA) of ChIP and input samples from Day 10 and Day 50 male fly heads. PCA was performed using genome-wide signal profiles for H3K4me1 and H3K27ac along with their corresponding inputs.

(D) H3K4me1 and H3K27ac enrichment at tissue-specific enhancers. Metaplots depict signal profiles on Day 10 and Day 50 over adult brain enhancers (*left*) and wing disc enhancers (*right*).

(E) Spearman correlation heatmaps of H3K4me1 (*left*) and H3K27ac (*right*) ChIP-seq samples from two biological replicates of male *Drosophila* heads collected at Day 10 and Day 50.

(F) Metaplots showing the distribution of H3K4me1 and H3K27ac ChIP-seq signals across differentially expressed genes (DEGs) in male *Drosophila* heads collected at Day 10 and Day 50. TSS and TES denote the transcription start site and transcription end site, respectively.

(G) Metaplots of H3K4me1 and H3K27ac ChIP-seq signals centered on the summits of common and age-differential H3K4me1/H3K27ac peaks identified between Day 10 and Day 50 in male *Drosophila* heads.

(H) Motif enrichment analysis of age-dependent ΔH3K4me1 and ΔH3K27ac peaks, with the predicted transcription factor (TF) binding to each motif indicated.

(I) Beeswarm boxplots illustrating RNA log_2 fold changes of genes associated with young > old ΔH3K27ac peaks in mouse forebrain NeuN+ neurons, compared with five size-matched random gene sets (R). "P" and "E/I" denote promoter and exon-intron peak locations, respectively.

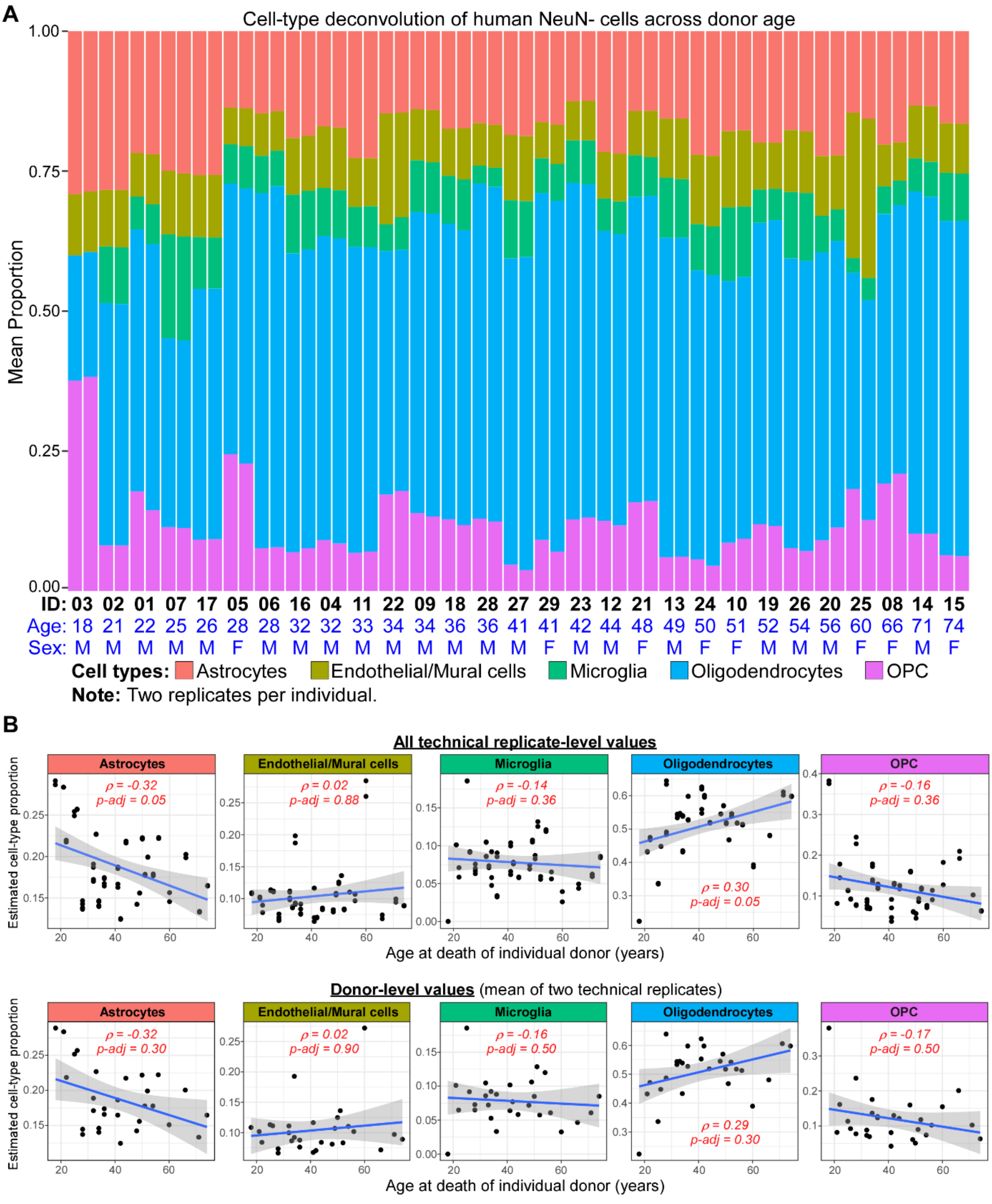

Figure S2. Cell-type deconvolution of human NeuN− cells across donor age.

A) Stacked bar plots showing the estimated proportions of astrocytes (salmon), endothelial/mural cells (olive), microglia (green), oligodendrocytes (blue), and oligodendrocytes precursor cells (OPC; magenta) in human NeuN− cell populations, estimated by deconvolution of bulk RNA-seq data. Each bar represents one technical replicate from an individual donor. Donor IDs, age at death, and sex are indicated below each bar; two technical replicates were analysed per individual.

B) Associations between estimated cell-type proportions and donor age. Each point represents an individual technical replicate (*top*) or the mean of two technical replicates per donor (*bottom*). Lines indicate fitted linear regressions and 95% confidence bands. Spearman correlation coefficients (ρ) and adjusted p-values are shown in each panel. Age is presented in years.

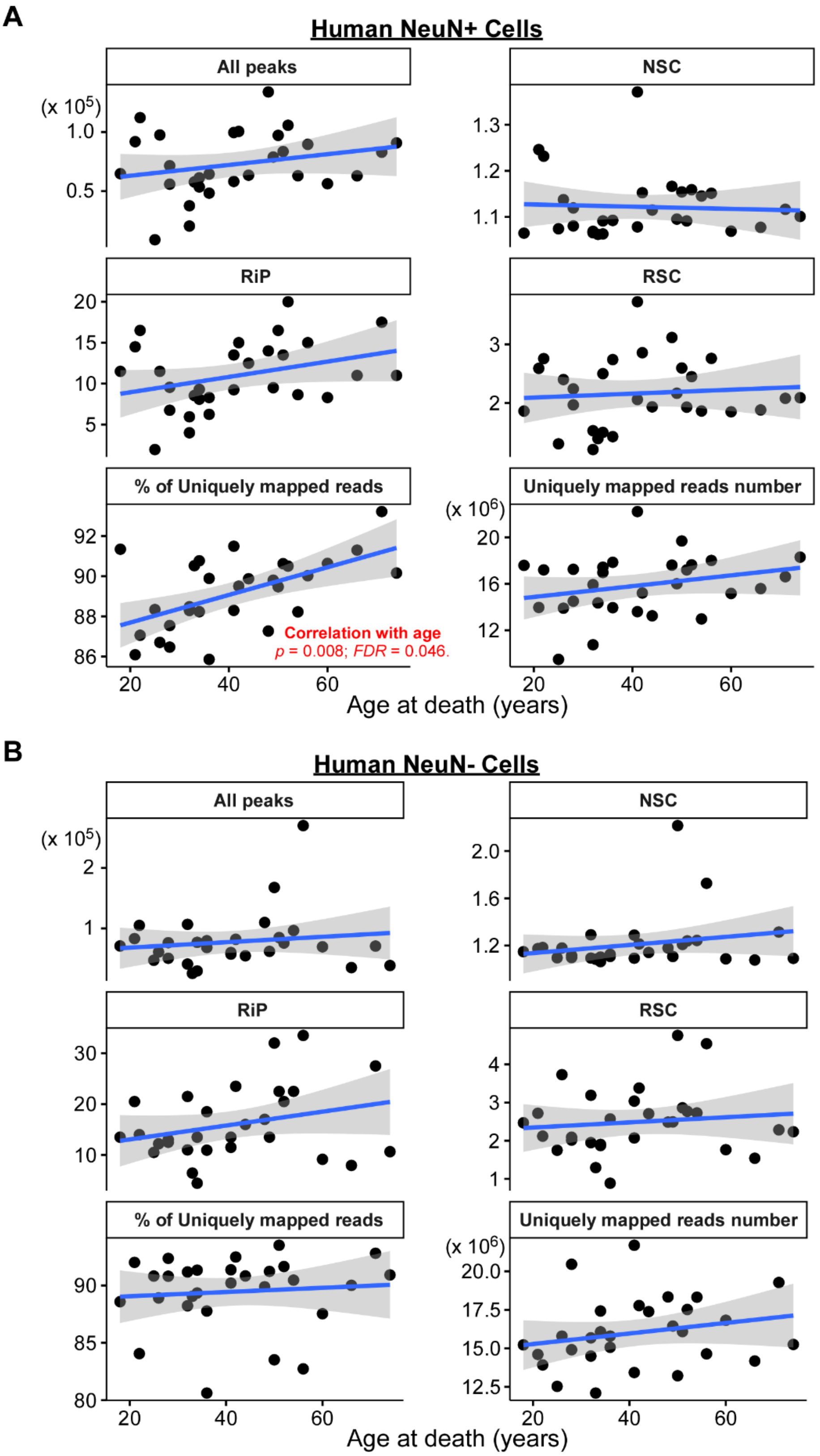

Figure S3. Association between donor age and ChIP-seq quality control metrics. Individual ChIP-seq quality control metrics from (A) NeuN+ and (B) NeuN− cells plotted against donor age. Only percent of uniquely mapped reads in NeuN+ neurons is modestly correlated with age of individuals.

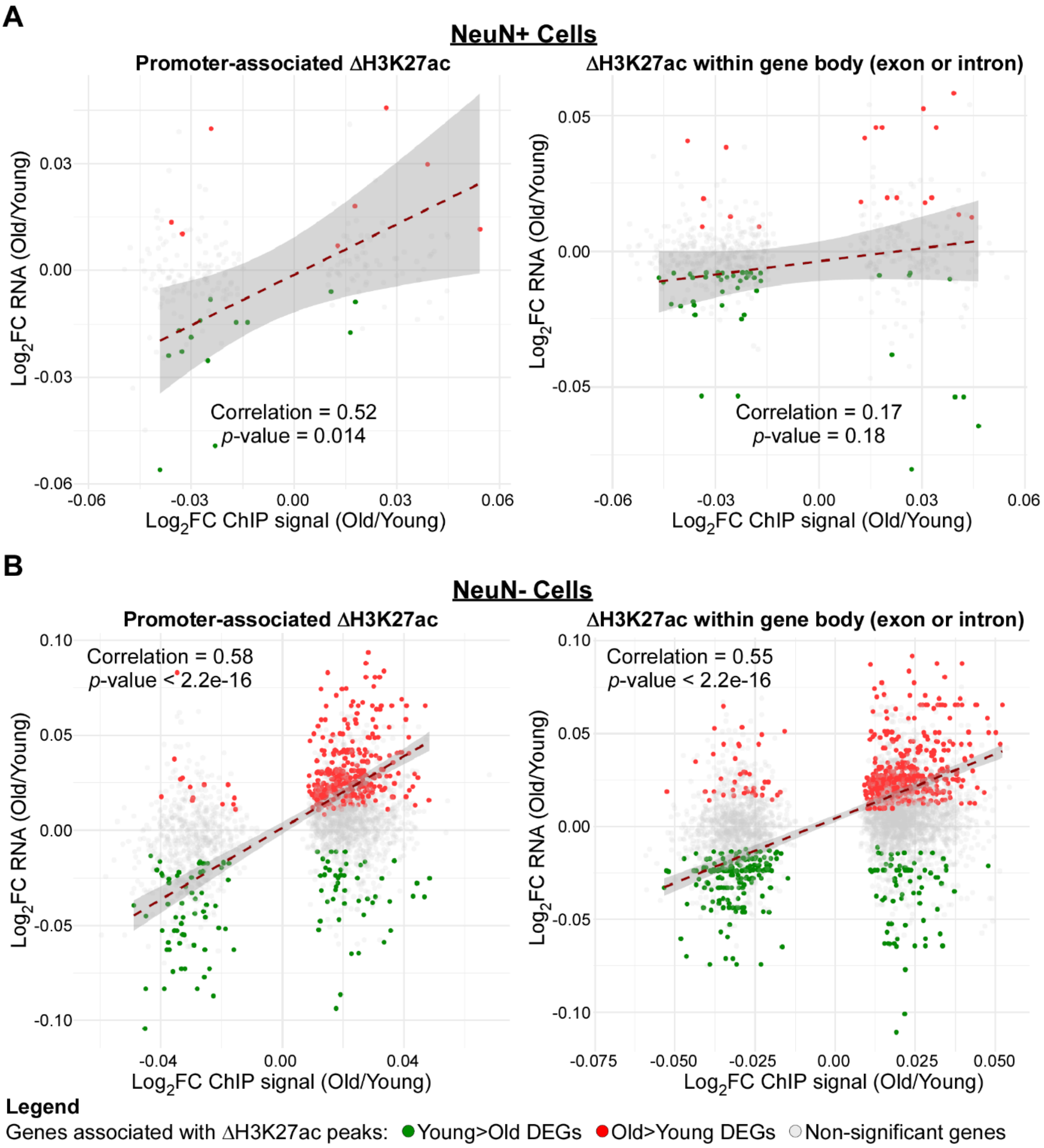

Figure S4. Effects of age-related changes in differential H3K27ac signals on the RNA expression of the associated genes in human NeuN+ and NeuN− cells.

(A–B) Scatterplots showing the relationship between RNA $\log_2$ fold change (old/young; y-axis) and ΔH3K27ac signal (Δ peaks with $p < 0.005$; x-axis) in aging human (A) NeuN+ and (B) NeuN− cells. Left, genes associated with ΔH3K27ac peaks at promoters; right, genes associated with ΔH3K27ac peaks located in exon-intron regions. Red and green points indicate DEGs ($p < 0.05$), while grey points represent non-DEGs. Genes associated with ΔH3K27ac peaks without detectable RNA expression were excluded. The dashed dark red line indicates linear regression fit, and the grey region represents the corresponding confidence interval.

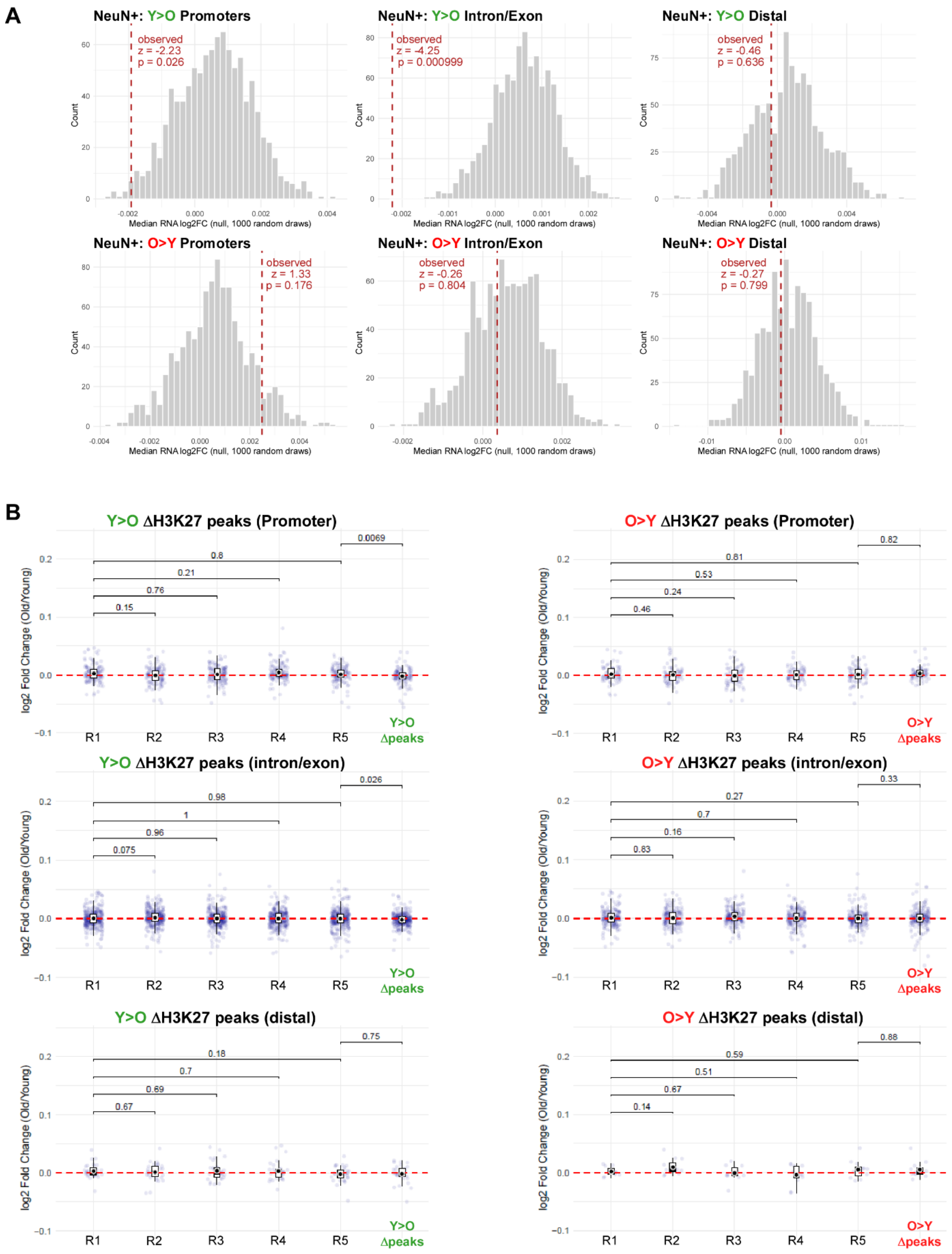

Figure S5A-B. Statistical comparison of expression patterns of genes associated with ΔH3K27ac peaks and matched random genes in human NeuN+ cells.

(A) Histograms showing the distributions of median RNA $\log_2$ fold-change values for 1,000 randomly sampled gene sets, each matched in size to the set of genes associated with

ΔH3K27ac peaks at distinct genomic loci in NeuN+ neurons. The reported *z* and *P* values indicate the standardized deviation of the observed median from the permutation distribution and its statistical significance, respectively. Red dashed lines indicate the observed median RNA $\log_2$FC for each gene set.

(B) Beeswarm boxplots showing RNA $\log_2$ fold changes for genes associated with (left) young > old and (right) old > young differential (Δ) H3K27ac peaks, compared with an equal number of randomly selected genes in NeuN+ cells from human frontal cortex. Genes were grouped according to the genomic location of the ΔH3K27ac peaks at promoters, exon-intron regions and distal enhancers inferred from neuronal Hi-C data.

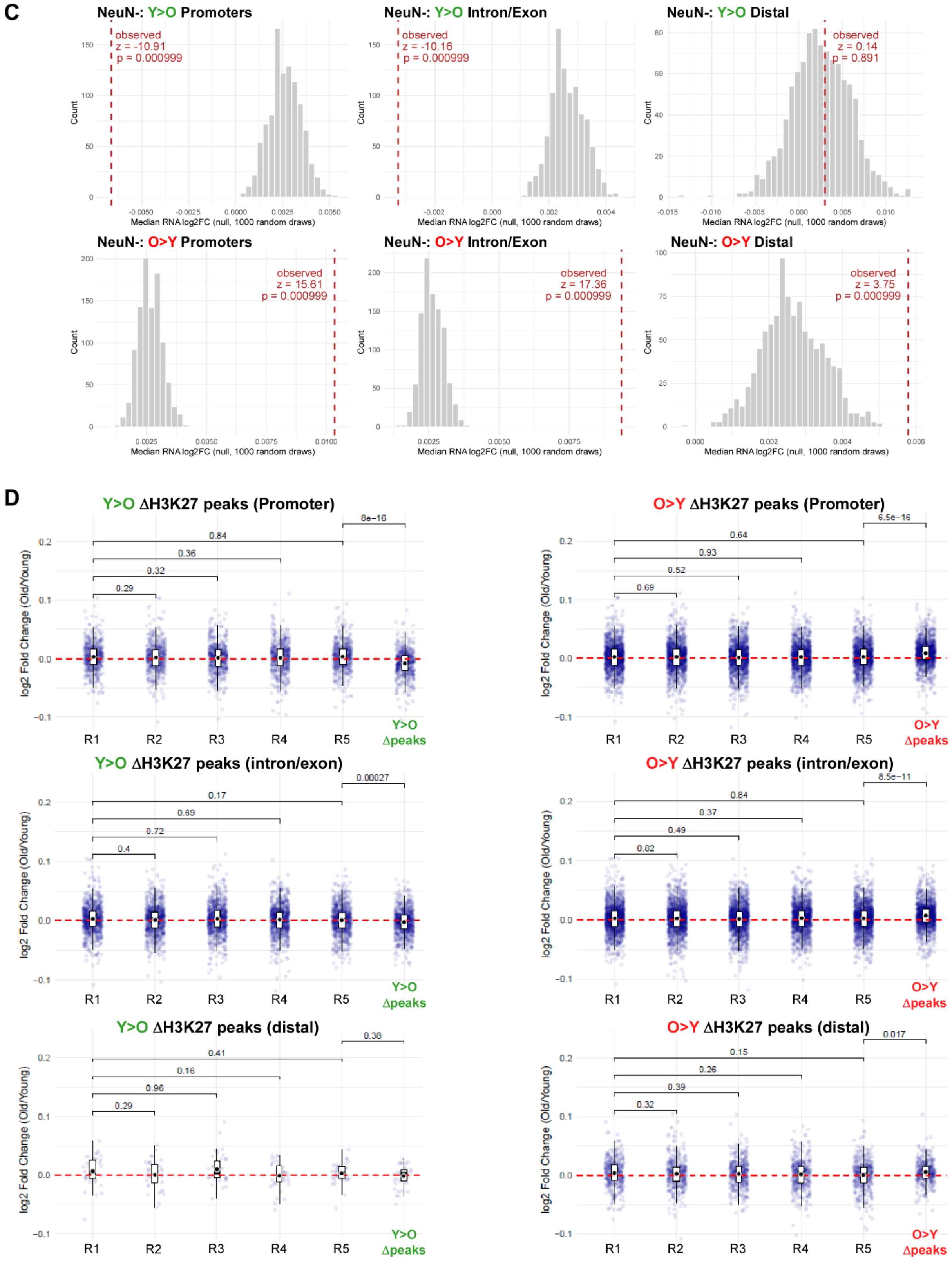

Figure S5C-D. Statistical comparison of expression patterns of genes associated with ΔH3K27ac peaks and matched random genes in human NeuN− cells.

(C) Histograms showing the distributions of median RNA log_2 fold-change values for 1,000 randomly sampled gene sets, each matched in size to the set of genes associated with

ΔH3K27ac peaks at distinct genomic loci in NeuN− cells. The reported *z* and *P* values indicate the standardized deviation of the observed median from the permutation distribution and its statistical significance, respectively. Red dashed lines indicate the observed median RNA $\log_2$FC for each gene set.

(D) Beeswarm boxplots showing RNA $\log_2$ fold changes for genes associated with (left) young > old and (right) old > young differential (Δ) H3K27ac peaks, compared with an equal number of randomly selected genes in NeuN− cells from human frontal cortex. Genes were grouped according to the genomic location of the ΔH3K27ac peaks at promoters, exon-intron regions and distal enhancers inferred from glial Hi-C data.

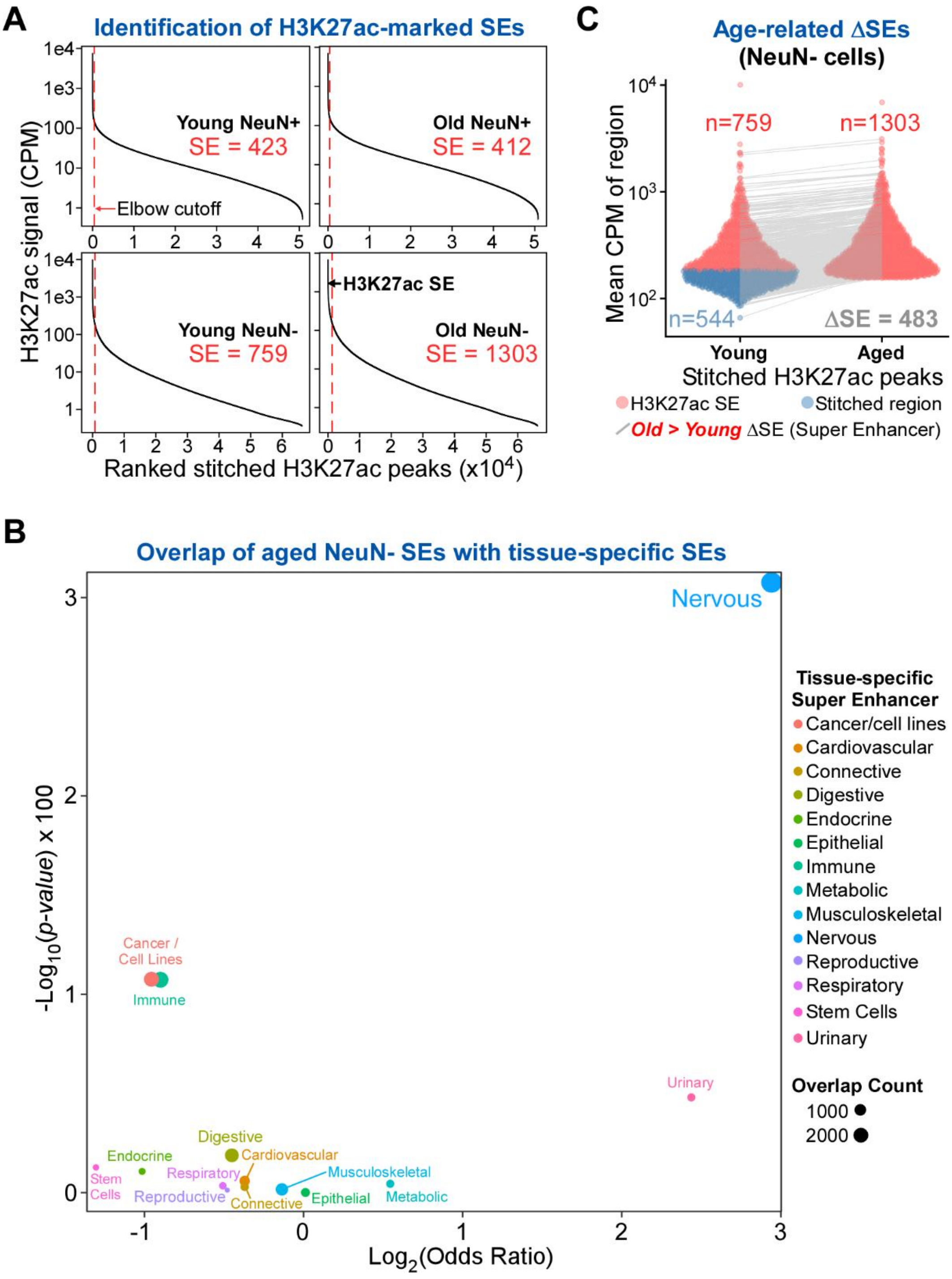

Figure S6 Age-dependent differential super-enhancers (SE) in NeuN− cells.

(A) Definition of H3K27ac-marked super-enhancers “SEs” in NeuN+ (*top*) and NeuN− (*bottom*) cells using the ROSE approach. Individual H3K27ac peaks were stitched into broader enhancer regions based on genomic proximity and ranked by normalized H3K27ac signal intensity. SEs are defined as enhancer regions above the inflection point of the curve, indicated by the red vertical dashed line.

(B) Overlap analysis of 1303 SEs identified in aged NeuN− cells with tissue-specific super-enhancers curated from the dbSUPER database revealed strong cell-type specificity.

(C) Beeswarm plot showing stitched H3K27ac regions and super-enhancers (SEs) in NeuN− cells from young and aged individuals. Differential SEs (ΔSE) are connected by grey lines. H3K27ac signal intensity for each stitched region was normalized as counts per million (CPM).

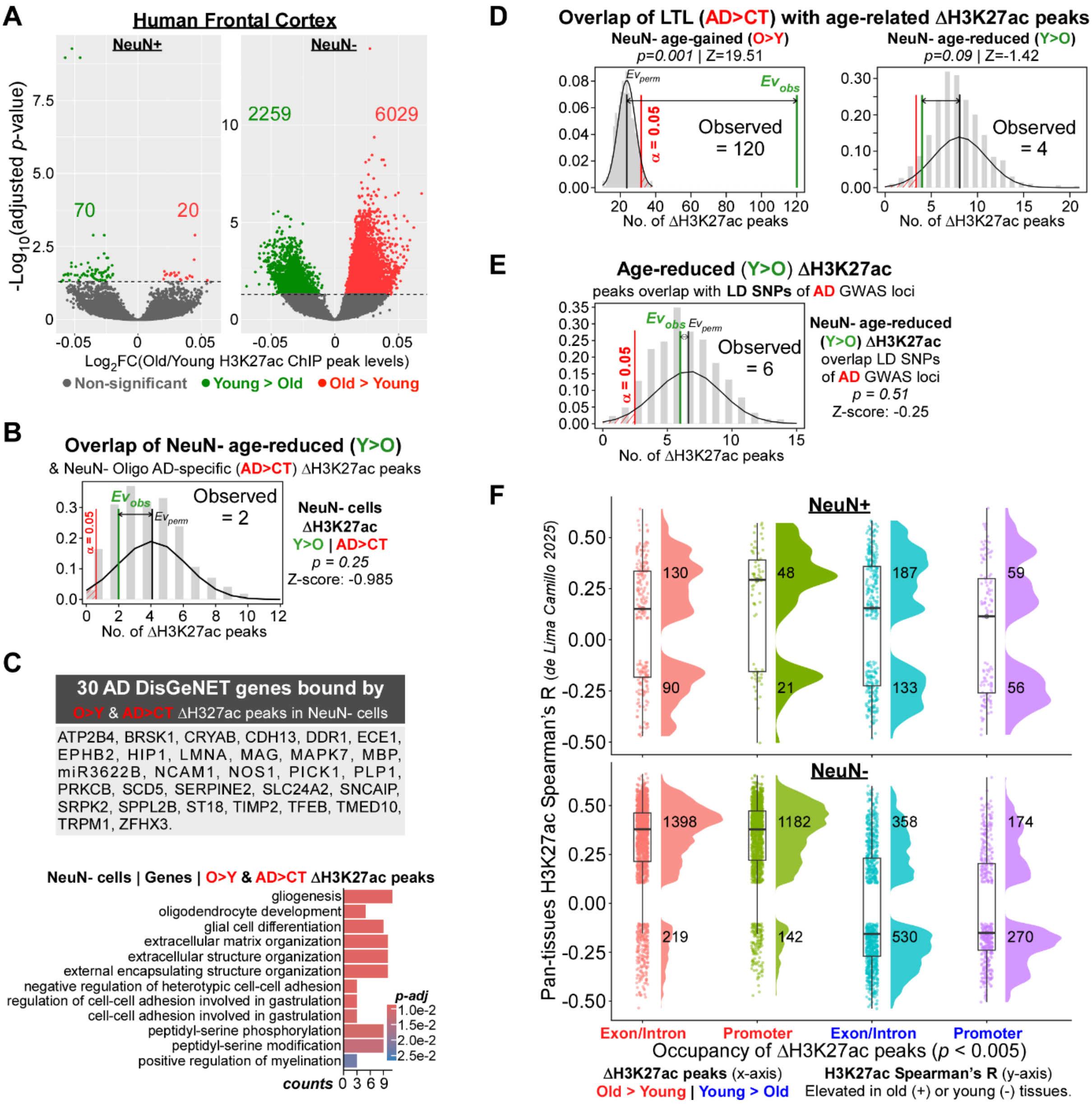

Figure S7. Multimodal analysis of ΔH3K27ac identified in NeuN− cells.

(A) Volcano plots showing ΔH3K27ac peaks (*p-adjusted* < 0.05) identified in NeuN+ neurons (left) and NeuN− cells (right) from human frontal cortex using a continuous age model. Only age-related ΔH3K27ac peaks from NeuN− cells were used for comparative analysis with AD datasets in Figure 2F onward.

(B) Permutation analysis of overlap between age-reduced ΔH3K27ac peaks in NeuN− cells and hyperacetylated H3K27ac peaks in AD NeuN− oligodendrocytes. Y-axis indicates the frequency distribution from 1000 permutations. Related to Figure 2I.

(C) (Top) List of curated AD genes from DisGeNET database associated with AD-specific and age-up ΔH3K27ac peaks in NeuN− cells. (Bottom) GO terms enrichment of genes bound by AD-specific and age-up ΔH3K27ac peaks in NeuN− cells. Related to Figure 2I.

(D) Permutation analysis of the overlap between age-related ΔH3K27ac peaks in NeuN− cells and ΔH3K27ac peaks identified in control versus AD lateral temporal lobe (LTL) samples. The y-axis indicates the frequency distribution of observing the overlap generated by permutation testing.

(E) Permutation analysis of the overlap between age-down (Y>O) ΔH3K27ac peaks in NeuN− cells and SNPs in linkage disequilibrium (LD) with AD GWAS lead variants. The y-axis represents the frequency distribution of overlap from 1000 permutations. Related to Figure 2K.

(F) Distribution of pan-tissue H3K27ac Spearman's R values for genes associated with age-dependent ΔH3K27ac peaks ($p < 0.005$) in human NeuN+ (top) and NeuN− (bottom) cells. Numbers indicate genes in each category. Red and blue denote age-up and age-down ΔH3K27ac peaks in frontal cortex cells, respectively (x-axis). Positive and negative R values indicate higher H3K27ac levels in old and young pan-tissue samples, respectively (y-axis). Related to Figure 2L.

2 | DESCRIPTION OF SUPPLEMENTAL DATA

https://drive.google.com/drive/folders/1kr5Xdn3TI4UBYVQ09f9bTafecr6WifHv

Table S1: *Drosophila* datasets.

(A) Differential expressed genes (DEGs) between D10 and 50 male fly heads.

(B) GO terms of DEG with statistically significant differences in their expression between Day 10 and 50 male fly heads.

(C) Number of DEGs assigned to concordant or discordant differential peaks.

(D) GO terms of DEGs associated with either (i) differential H3K27ac peaks less than -5kb of their TSS (D50>D10) or (ii) H3K4me1 peaks in gene body (D50>D10).

Table S2: Mouse datasets.

(A) Metadata of H3K27ac ChIP-seq of NeuN+ neurons from 3- & 24-months old mice [6].

(B) Differential H3K27ac peaks (3 > 24 months) in mouse NeuN+ cells.

(C) Differential H3K27ac peaks (24 > 3 months) in mouse NeuN+ cells.

(D) GO terms of genes with differential H3K27ac at promoter (Y>O) & aged-dependent alterations in mRNA expression (Y>O or O>Y).

(E) GO terms of genes with differential H3K27ac in exon-intron (Y>O) & aged-dependent alterations in mRNA expression (Y>O or O>Y).

(F) Enriched motifs and predicted transcription factors at ΔH3K27ac peaks (Y>O) associated with concordant and discordant mRNA expression (Y>O or O>Y).

Table S3: Human datasets.

(A) Metadata & QC metrics of neurological healthy controls from (phs002487.v1.p1) [10].

(B) Age-gained and age-reduced differential H3K27ac peaks in NeuN+ cells

(C) Age-gained and age-reduced differential H3K27ac peaks in NeuN− cells

(D) List of age-associated differential expressed genes (DEGs) in NeuN+ cells

(E) List of age-associated differential expressed genes (DEGs) in NeuN− cells

(F) Top 20 enriched GO terms of genes associated with Y>O differential H3K27ac peaks at exon/intron and Y>O expression in NeuN+ cells

(G) Enriched GO terms of genes associated with age-related differential H3K27ac peaks and expression changes in NeuN− cells

(H) Enriched motifs and their predicted transcription factors (TFs) at various differential H3K27ac peaks in NeuN+/- cells

(I) List of super enhancers defined by stitched H3K27ac peaks in different age groups of NeuN− & NeuN+ cells with their respective CPM scores

(J) GO terms of protein-coding genes with age-related ΔH3K27ac peaks and expression changes overlapping with AD DEGs

(K) Overlap of ΔH3K27ac peaks elevated in aged NeuN− cells (age-gained) and AD NeuN− oligodendrocytes (AD-specific)

(L) GO terms of genes associated with both O>Y & AD>CT ΔH3K27ac peaks in NeuN− cells

(M) Enriched motifs & predicted TFs for overlapping age-gained and AD-specific ΔH3K27ac peaks with concordant gene expression

(N) Summary of AD GWAS lead SNPs and the number of LD proxies

(O) Overlap of differential H3K27ac peaks (O>Y) in NeuN− cells with LD SNPs of AD GWAS loci
